# The MicroRNA *miR-277* Controls Physiology and Pathology of the Adult *Drosophila* Midgut by Regulating the Expression of Fatty Acid β-Oxidation-Related Genes in Intestinal Stem Cells

**DOI:** 10.3390/metabo12040315

**Published:** 2022-03-31

**Authors:** Lisa Zipper, Sai Batchu, Nida Hatice Kaya, Zeus Andrea Antonello, Tobias Reiff

**Affiliations:** 1 Institute of Genetics, Department of Biology, The Faculty of Mathematics and Natural Sciences, Heinrich-Heine-Universität Düsseldorf, 40225 Düsseldorf, Germany; lisa.zipper@hhu.de; 2 Cooper Medical School, Rowan University, Camden, NJ 08102, USA; batchu94@rowan.edu (S.B.); antonello@rowan.edu (Z.A.A.); 3 Institute for Zoology and Organismic Interactions, Department of Biology, The Faculty of Mathematics and Natural Sciences, Heinrich-Heine-Universität Düsseldorf, 40225 Düsseldorf, Germany; nida.kaya@hhu.de; 4 Cooper University Hospital, Cooper University Health Care, Cooper Medical School, Rowan University, Camden, NJ 08102, USA

**Keywords:** intestinal stem cell, metabolism, fatty acid oxidation, *Drosophila*, midgut

## Abstract

Cell division, growth, and differentiation are energetically costly and dependent processes. In adult stem cell-based epithelia, cellular identity seems to be coupled with a cell’s metabolic profile and vice versa. It is thus tempting to speculate that resident stem cells have a distinct metabolism, different from more committed progenitors and differentiated cells. Although investigated for many stem cell types in vitro, in vivo data of niche-residing stem cell metabolism is scarce. In adult epithelial tissues, stem cells, progenitor cells, and their progeny have very distinct functions and characteristics. In our study, we hypothesized and tested whether stem and progenitor cell types might have a distinctive metabolic profile in the intestinal lineage. Here, taking advantage of the genetically accessible adult *Drosophila melanogaster* intestine and the availability of ex vivo single cell sequencing data, we tested that hypothesis and investigated the metabolism of the intestinal lineage from stem cell (ISC) to differentiated epithelial cell in their native context under homeostatic conditions. Our initial in silico analysis of single cell RNAseq data and functional experiments identify the *microRNA miR-277* as a posttranscriptional regulator of fatty acid β-oxidation (FAO) in the intestinal lineage. Low levels of *miR-277* are detected in ISC and progressively rising *miR-277* levels are found in progenitors during their growth and differentiation. Supporting this, *miR-277-*regulated fatty acid β-oxidation enzymes progressively declined from ISC towards more differentiated cells in our pseudotime single-cell RNAseq analysis and in functional assays on RNA and protein level. In addition, in silico clustering of single-cell RNAseq data based on metabolic genes validates that stem cells and progenitors belong to two independent clusters with well-defined metabolic characteristics. Furthermore, studying FAO genes in silico indicates that two populations of ISC exist that can be categorized in mitotically active and quiescent ISC, of which the latter relies on FAO genes. In line with an FAO dependency of ISC, forced expression of *miR-277* phenocopies RNAi knockdown of FAO genes by reducing ISC size and subsequently resulting in stem cell death. We also investigated *miR-277* effects on ISC in a benign and our newly developed CRISPR-Cas9-based colorectal cancer model and found effects on ISC survival, which as a consequence affects tumor growth, further underlining the importance of FAO in a pathological context. Taken together, our study provides new insights into the basal metabolic requirements of intestinal stem cell on β-oxidation of fatty acids evolutionarily implemented by a sole microRNA. Gaining knowledge about the metabolic differences and dependencies affecting the survival of two central and cancer-relevant cell populations in the fly and human intestine might reveal starting points for targeted combinatorial therapy in the hope for better treatment of colorectal cancer in the future.

## 1. Introduction

The ability of organisms to maintain their internal conditions in balance requires energy uptake. An organism’s body is considered to be homeostatic while it constantly compensates for fluctuating wear and tear, injuries, changes in environment, and energetical in- and effluxes. To cope with these unpredictable conditions, evolution generated plastic and context-dependent ways to adapt to changes in e.g., nutrients availability. Nutrient-related adaptations go down until the cellular level, affecting the way each cell utilizes resources of energy. Growth, division, and differentiation are genetically encoded processes and are thought to adapt their gene expression in a plastic and context-dependent manner [1,2,3,4].

Prime candidates for such a complex regulation of gene expression of entire pathways and networks are microRNAs, which regulate approximately half of the transcriptome [1,2]. Indeed, apart from established roles in key processes such as cytoskeletal dynamics, cell migration, stemness, and metabolic phenotype [3,4,5,6], microRNAs have been implicated in the control of glucose and lipid metabolism [7].

In adult *Drosophila melanogaster* females, lipid metabolism is upregulated in a regionalized subpopulation of differentiated intestinal cells upon mating to sustain egg production [5,6,7]. Such regional differences of intestinal cell types have not only been reported regarding metabolism, but localization also seems to be connected with stem cell (SC) identity and proliferation behavior [8,9,10]. Together, this leads to the intriguing question on which metabolic phenotype intestinal stem cells (ISC) rely and whether their metabolism is involved in SC behavior.

In general, in vivo data on SC metabolism is scarce owing to the lack of proper genetic sensors and tools. Here, we employed the fruit fly *Drosophila melanogaster,* taking advantage of its exhaustive genetic toolbox to study SC metabolism in an adult organism during physiological homeostasis and in the pathological context of tumoral growth. Briefly, the fly intestine harbors around one thousand multipotent ISC, showing regional differences in proliferation behavior and cellular anatomy [8,9,11,12,13]. ISC are able to self-renew, but mainly divide asymmetrically to generate two types of precursor cells: postmitotic enteroblasts (EB) capable of differentiation to absorptive enterocytes (EC) and enteroendocrine precursor cells (EEP) that perform a singular symmetric division giving rise to two enteroendocrine cells (EE) [11,12,14].

For a long time, it was generally assumed that SC harbor immature mitochondria allowing no or limited ATP production through oxidative phosphorylation (OXPHOS). Recent and widespread literature on SC metabolism has provided new insights, revealing that the metabolic phenotype of SC varies depending on species, age, and developmental stage, as well as tissue localization [15]. Studies of embryonic, mesenchymal, neural, and induced pluripotent SC also show that their metabolic phenotype varies from glycolysis, over β-oxidation of fatty acids (FAO) to OXPHOS as an energy source [15,16], suggesting that there is no SC metabolism per se. An important commonality of these studies is that the vast majority was performed in vitro where the sheer availability of oxygen and metabolites in culture media might affect metabolic (re-)programming of cultured SC.

In the adult *Drosophila* intestine, a few studies hint to metabolic pathways for energy allocation in ISC and how this influences homeostatic processes. In aging ISC, energy supply from nutrient stores is reduced, negatively affecting tissue homeostasis in old flies [17]. A few in vivo studies shed first light on ISC metabolism. Schell and colleagues demonstrated a requirement of mitochondrial metabolism in ISC for intestinal homeostasis in *Drosophila* and intestinal organoids, which suggests OXPHOS as a possible energy source for *Drosophila* ISC.

A second study suggests a direct link between ISC metabolic state and nutrient availability. By manipulating a key enzyme of the hexosamine biosynthesis pathway, Mattila and colleagues were able to shift the balance between OXPHOS and glycolysis, linking nutrient content directly to ISC proliferation [18,19]. Recent work from the Edgar lab confirms metabolic changes in EB while differentiating and describes that EGFR signaling increases glycolysis, FAO, and mitochondrial biogenesis [19,20]. In addition, input from EGFR signaling was shown to specifically affect EB survival, supporting the idea of different metabolism and thus nutrient requirements of ISC and EB [21]. Downregulation of key metabolic enzymes for glycolysis and OXPHOS does not affect ISC numbers, suggesting that both metabolic pathways are dispensable for basal metabolism controlling ISC survival [18,20,22]. However, genetic manipulations in both studies were performed using the *esg-Gal4* driver line which is active in ISC and EB, thus not allowing discrimination between metabolic requirements in stem or maturing precursor cells [22].

Here, building on in silico data from clustering of metabolic genes and pathways in *Drosophila* ISC, we set out to functionally identify and characterize the role of FAO genes in ISC metabolism in vivo. Investigating physiology and pathology of genetic manipulations of FAO enzymes, we add to the growing knowledge of metabolic essentials of intestinal stem cells.

## 2. Results

### 2.1. Identification of miR-277 as a Regulator of Cellular Metabolism

**Figure 1:**
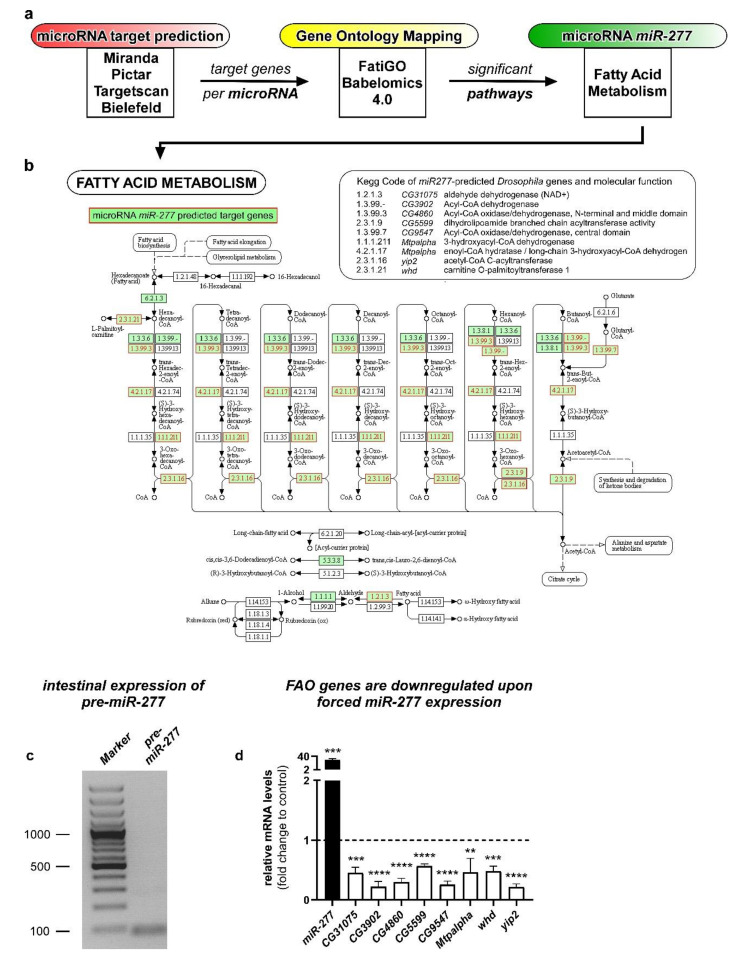
Workflow of the identification process for Gene Ontology Networks from microRNA target gene prediction and subsequent Gene Ontology mapping and proof of actual target gene regulation by *miR-277*. (**a**) Putative target gene lists for different miRNAs were obtained and analyzed from four microRNA prediction algorithms. Target genes that showed up in at least three out of four prediction algorithms were subjected to FatiGO prediction for Gene Ontology networks on Babelomics 4.0 servers (Barcelona, Spain) and resulting GO-terms (Gene Ontology) with a *p*-value *p* < 0.05 were considered. (**b**) Summary of predicted *miR-277* target genes involved in fatty acid metabolism: shown is the involvement of *miR-277* regulated genes in KEGG nomenclature with red lettering and green background. The table (top-right) lists all targeted genes from the KEGG pathway prediction for fatty acid metabolism, the according *Drosophila melanogaster* genes, and functions. KEGG involvement image modified, copyright by Kanehisa Laboratories. (**c**) PCR reaction using specific primer sets for the *pre-miR-277* reveal *miR-277* on adult *Drosophila* midgut cDNA; (**d**) relative mRNA levels of predicted *miR-277* target genes involved in fatty acid metabolism were decreased in whole guts upon forced expression of *UAS-miR-277* in EC using a *Mex^ts^-Gal4* driver (*n* = 3; unpaired *t*-test: ** *p* < 0.01, *** *p* < 0.001, **** *p* < 0.0001).

Changing cell metabolism requires fundamental adaptations of genetic networks and gene expression, which is why we aimed at microRNAs regulating such complex genetic networks. Approximately half of the transcriptome is regulated by microRNAs, which makes them a perfect candidate for the control of such metabolic networks [23,24]. Taking advantage of in silico resources [8,13], putative target gene lists from four different miRNA prediction algorithms were compared (Figure 1a). Predicted target genes of individual miRNAs showing up in at least three out of four miRNA prediction algorithms were then subjected to Gene Ontology Mapping using FatiGO Babelomics 4.0 (http://www.babelomics.org/, accessed on 5 November 2021).

To our surprise, from eight investigated microRNAs, only *microRNA miR-277* showed a significant enrichment of a set of predicted target genes all mapping to metabolic pathways (Figure S1). Analyzing and assigning this gene set using KEGG pathways revealed that eight of the *miR-277* regulated genes possess enzymatic functions in fatty acid metabolism (Figure 1b) and a known role in branched chain amino acid catabolism [25]. Furthermore, *miR-277* was linked to lipid metabolism in *Aedes aegypti* before [26].

The direct regulation of predicted target genes by *miR-277* was investigated using quantitative real- time PCR. Relative mRNA levels of predicted target genes were decreased in whole guts of adult *Drosophila* upon forced expression of *miR-277* in EC (Figure 1d), indicating that all eight target genes involved in fatty acid metabolism are in fact regulated by *miR-277*. β-oxidation of fatty acids (FAO) provides an important energy source by degrading fatty acids fueling the citrate cycle with acetyl-CoA (Figure 1b). Together with our previous data showing activation of lipid uptake in the posterior midgut upon mating [6], we aimed to investigate the role of *miR-277* and FAO in intestinal progenitors.

### 2.2. miR-277 Is Expressed in Differentiating EB and EC in the Adult Drosophila Intestine

**Figure 2:**
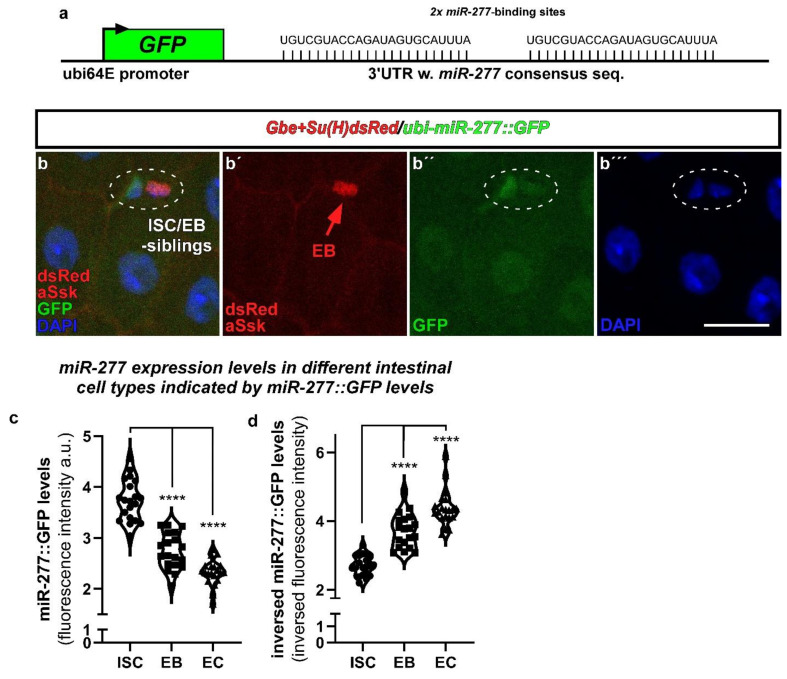
*miR-277*-expression in ISC, EB and EC revealed by *miR-277::GFP* sensor flies in the R5 region of the posterior midgut. (**a**) miR-277-expression in R5 regions of adult posterior midguts was analyzed using a transgenic sensor for *miR-277*. The sensor is expressed ubiquitously by ubi-promoter sequences and consists of *miR-277* consensus sequences fused to the coding sequence of GFP. Thus, raised *miR-277* levels directly reduce GFP signals. (**b**–**b’’’**) Flies carrying the *miR-277* sensor were crossed with flies carrying the Notch-activity reporter *Gbe+Su(H)dsRed* labelling EB. After seven days at 25 °C, images were taken with fixed 488 nm laser settings to enable comparison of GFP-intensity (**b’’**). *Ubi-miR-277::GFP* flies reveal a significant decrease in GFP-signal in EB and epithelial EC (identified by big nuclear size and aSsk staining of septate junctions) compared to GFP signal detected in ISC. (**c**,**d**) Quantification of GFP-fluorescence intensity of ISC, EB and EC corrected to control *ubi-GFP* flies ((**c**); see materials and methods; *n* = 20, 19, 20; ANOVA, **** *p* < 0.001) and numerical inversion for comprehensibility (**d**). (Scale bar is 10 µm).

Intrigued by these results, we tested for *miR-277* expression in the posterior region of the female adult *Drosophila* intestine and found pre-miRNA-277 expressed in the adult midgut by PCR (Figure 1c). Therefore, we used *miR-277* sensor flies containing a transgene consisting of *miR-277* consensus sequences fused to a sequence coding for GFP (Figure 2a). Presence of *miR-277* results in mRNA degradation and therefore reduced GFP-levels compared to control flies lacking these consensus sequences [27,28]. Crossing these flies with a reporter for Notch-signaling activity (*NRE-mcherry or Gbe+Su(H)dsRed,* marking EB) enabled us to decipher ISC from EB and epithelial EC and EE (Figure 2b,b’’’) [11,29].

ISC and EB usually occur as duplets (Figure 2b) and maturing EB separate from their mother ISC to differentiate into EC [3,21,30]. We addressed *miR-277* expression levels by measuring GFP fluorescence in ISC, EB, and EC. Presence of endogenous *miR-277* leads to degradation of GFP encoding mRNA and thus fluorescence intensity. Highest GFP fluorescence was measured in ISC (Figure 2b,c), reflecting low endogenous *miR-277* levels (Figure 2d). Differentiating EB and terminally differentiated EC have significantly lower GFP-levels, suggesting higher *miR-277* levels (Figure 2c,d), which in turn might reflect less FAO activity. We validated the reactivity of the *miR-277* sensor flies to changes in *miR-277* expression by crossing *miR-277::GFP* flies with overexpression (*UAS-miR-277*) or knockdown of *miR-277* using an miR-277 sponge genetic construct (*UAS-miR-277-sp*, Figure 6e). MicroRNA sponges contain multiple complementary binding sites to the seed sequence of a microRNA of interest (Figure 6e) and reduce endogenous microRNA levels in both flies and human cell culture [31,32]. Upon overexpression of *miR-277* in ISC/EB, *miR-277::GFP* intensities were decreased, while knockdown of *miR-277* resulted in increased GFP intensities (Figure S4f–i). These results prove that the sensor is reflecting *miR-277* expression levels and underlines endogenous *miR-277* expression in ISC/EB. Next, we set out to determine FAO gene transcription in existing scRNAseq datasets of intestinal cell types [13].

### 2.3. miR-277 Target Gene Expression in Reconstructed Intestinal Lineage Trajectories from scRNAseq

To investigate the expression of *miR-277* target genes and possible metabolic differences between ISC/EB and differentiated progeny, we used the updated 2019 single-cell sequencing dataset [13] to perform a cell clustering and lineage reconstruction based only on metabolic genes [33,34]. To infer cell lineage, we used only ISC/EB, differentiating EC, EC (anterior (aEC), mid (mEC) and posterior EC (pEC)), and EEs, and excluded all other cell types following a previous analysis [13].

**Figure 3:**
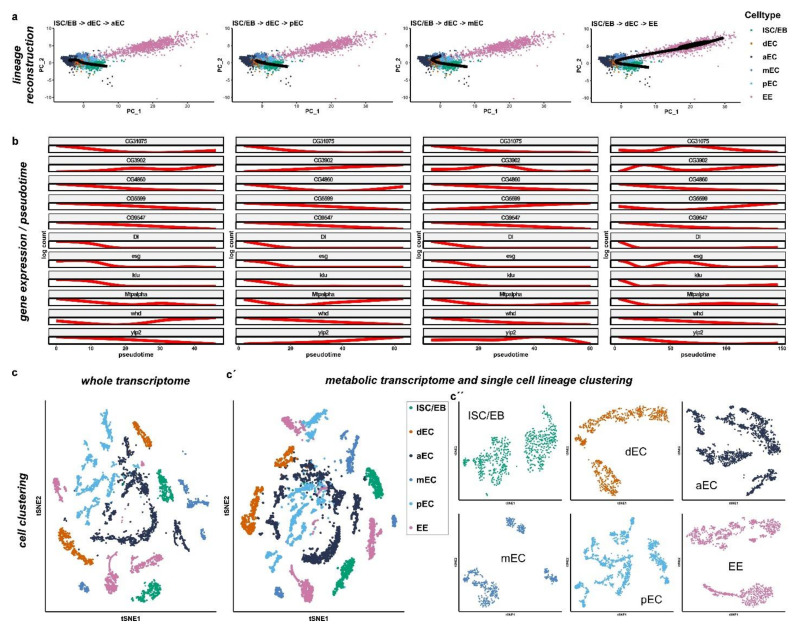
Metabolic transcriptome analysis of single-cell sequencing data from the cell atlas of the *Drosophila* midgut. (**a**) Cell lineages based on metabolic genes expression were inferred using Slingshot. Four lineages were constructed starting from ISC/EB and ending in each differentiated cell type (anterior EC (aEC), middle EC (mEC), posterior EC (pEC), and EE). (**b**) Plot of gene expression as a function of pseudotime for each lineage. Target genes of *miR-277* are shown together with the known markers of ISC and EB (*esg, Dl, klu* and *pros*). (**c**–**c’’**) Comparison of whole transcriptomic (**c**) and only metabolic genes (**c’**–**c’’**) cell clustering with correspondent activity score. (**c’**,**c’’**) Metabolic cell clusters separated by cell type (**c’**) and single cell lineage clustering (**c’’**) as previously described.

Four main lineages were reconstructed: (i) ISC/EB→differentiating EC (dEC)→anterior EC (aEC), (ii) ISC/EB→dEC→mid EC (mEC), (iii) ISC/EB→dEC→posterior EC (pEC) and (iv) ISC/EB→EE (Figure 3a). ISC/EB and each EC subtype (aEC, mEC and pEC) showed clustering with a small dispersion indicating a homogenous metabolic profile from anterior to posterior EC (Figure S2a–c). EE instead showed the highest dispersion/metabolic heterogeneity in accordance with a high variety of EE subtypes [35] (Figure 3a). Pseudotime analysis of predicted *miR-277* target genes indicates that most genes of the FAO pathways predicted to be regulated by *miR-277* are progressively inhibited in dEC towards terminally differentiated EC lineages and in the lineage specification of EE as well (Figure 3b).

In detail, the reconstructed lineage towards pEC shows that FAO metabolic genes *CG31075, CG4860, CG5599, CG9547,* and *whd* diminish, while *yip2* and *CG3902* progressively increase (Figure 3b). *Mtpalpha* diminishes during the differentiation to then increase again in the terminally differentiated pEC (Figure 3b). In the reconstructed lineage towards anterior EC, the expression profile is similar to the posterior midgut with the difference of *yip2*, which progressively increases, and *whd*, which decreases during differentiation to then increase in terminally differentiated anterior EC, similarly to *Mtpalpha* for pEC (Figure 3b). In the mid midgut, reconstructed lineages also show similar patterns to the pEC, with the exception of *CG5599* which increases progressively, *CG3902* which initially increases in the process of differentiation to then diminish in terminally differentiated mEC, and *yip2* which increases initially and then diminishes. Although few FAO genes seem to be more expressed in differentiated cells or seem to be fluctuate in the process of differentiation, altogether these data show that FAO genes expression levels diminish towards differentiated cells.

This is in accordance with our in vivo data showing that *miR-277* levels increase towards EC fate (Figure 2c,d) and further indicates that *miR-277* post-transcriptionally represses FAO genes (Figure 1d). To confirm our in silico pseudotime analysis, we investigated flies carrying a GFP-tagged CG9547 fusion protein under control of endogenous regulatory sequences (Figure S5a). We found highest CG9547 protein levels in Notch ligand Delta (Dl) positive ISC (Figure 5d and Figure S5b–b’’’). Using specific markers for intestinal cells [5,11,12,21,29,36], we were able to validate our pseudotime analysis of an expression decline for CG9547 towards epithelial EC cell fates on protein level (Figure 5d). In addition, we could prove that CG9547 protein levels are reduced upon overexpression of *miR-277* (Figure S5e,e’,h) or knockdown of *CG9547* by RNAi (Figure S5g,g’,h) compared to controls (Figure S5d,d’,h). Upon knockdown of *miR-277* using *miR-277-sp* CG9547 protein levels increase (Figure S5f,f’,h). This observation supports our results showing endogenous expression and regulation of the predicted target genes involved in FAO by *miR-27* 7 in ISC/EB.

The finding of CG9547 FAO enzyme expression in ISC and its regulation by *miR-277* strongly underlines our metabolic analysis from scRNAseq. Our observations suggest that fatty acids in ISC are used for energy production through FAO and might rather be used for membrane build-up in dEC and EC in an anabolic way. Together with the finding that FAO regulating *miR-277* levels are low in ISC, we wanted to investigate the hypothesis whether fatty acids might be used differently between ISC and EB.

### 2.4. FAO defines Differences among Intestinal Stem Cells and Enteroblasts

**Figure 4:**
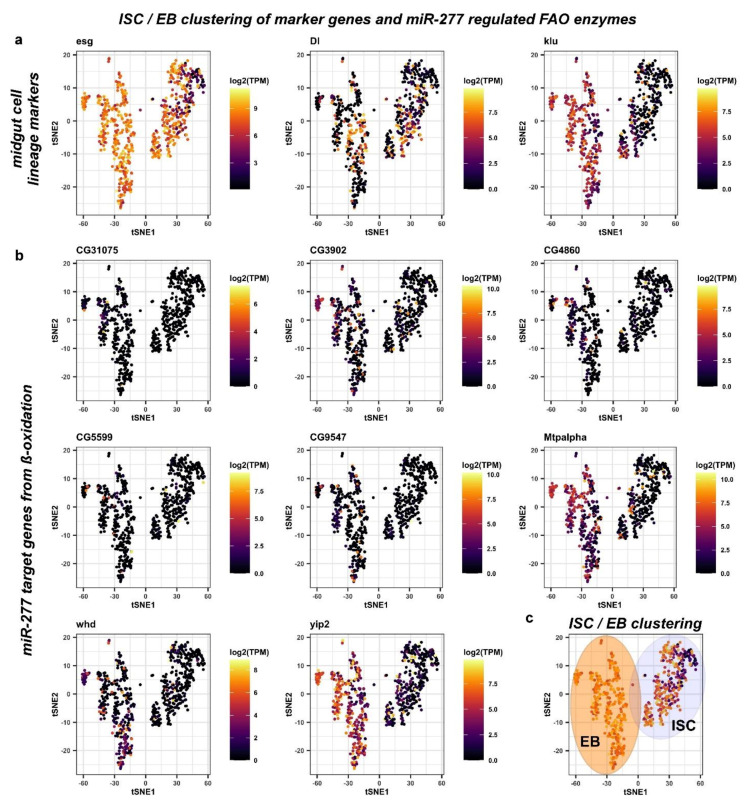
Analysis of midgut progenitor cell markers and *miR-277* target genes from ß-oxidation in metabolic cell clusters of ISC/EB. (**a**) Two distinct ISC/EB clusters in the metabolic cell clusters were identified being positive for *esg* expression. The ISC marker *Dl* is expressed in cells of both clusters that are negative for expression of the EB marker *klu*. (**b**) Analysis of *miR-277* target genes from FAO in metabolic cell clusters of ISC/EB (**c**) ISC/EB clusters can be subdivided into two distinct metabolic clusters, ISC (grey circle) and EB (orange circle) analyzing expression of *Dl* and *klu* marker genes.

Stem cells and progenitor cells, although both undifferentiated progenitor cell types, are fundamentally different. In the adult *Drosophila* intestine, ISC are the only cell type capable of self-renewal, while EB are postmitotic and committed towards EC differentiation. EB are a transient but discrete cell type capable of delaying terminal differentiation for sustained periods of time. During differentiation, EB obtain functional mitochondria and endoreplicate [3,5,19,20,21,30]. To investigate whether expression of genes involved in metabolic pathways in ISC and EB have differences reflecting their functional differences, we subdivided the ISC/EB cluster obtained in the principal component analysis of metabolic genes (Figure 3c–c’’) using previously described cell type specific markers such as *Dl* (ISC), *esg* (ISC+EB), *klu* (EB) and *pros* (EE) [5,11,12,21,29,36] (Figure 4a).

*Escargot (esg)* marks both ISC and EB, as previously demonstrated [3,37,38]. However, *esg* has been shown to overlap with EE [3,37,38,39]. First, we observed and excluded a very small number of *pros^+^* cells within the *esg^+^* ISC/EB population from the analysis (Figure S3a). The Notch ligand *Delta (Dl)* has been shown to be a sufficient but not necessary marker of ISC *per se*, as it is only necessary during stem cell division for EB cell fate determination by Delta/Notch signaling [1,12,21,31,32]. In EB, the Notch target gene *klumpfuss (klu)* has been shown to be a sufficient and necessary marker for EB, preventing the EE fate and committing towards the EC fate [21,36]. We hence initially defined ISC as ‘*esg^+^, Dl^+^, klu^−^,* and *pros^−^*’, and EB as *‘esg^+^, Dl^−^, klu^+^,* and *pros^−^’* (Figure 4a). Using these criteria for mapping, *miR-277* targeted FAO enzymes are found to be expressed in ISC/EB (Figure 4b) and two distinct populations of ISC and EB can be distinguished (Figure 4c). Despite that, we found a high variety of genes contradicting only one ISC population in terms of metabolic gene expression.

**Figure 5:**
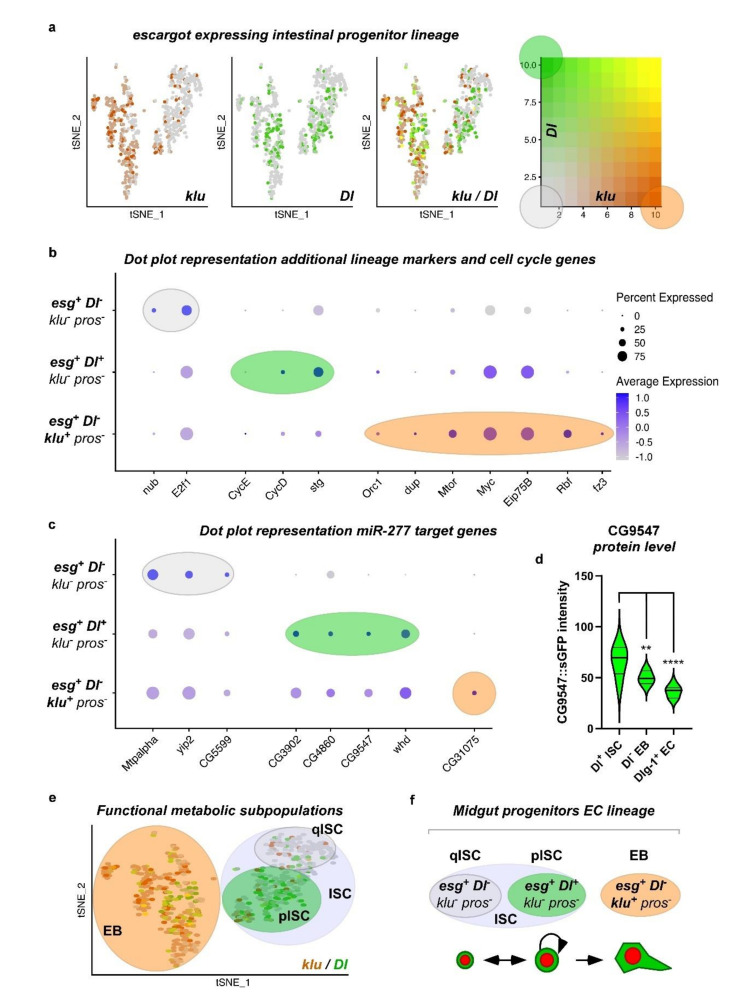
Subclustering of ISC/EB using combined expression of *esg*, *Dl*, *klu,* and *pros* identify quiescent and proliferating ISC within the ISC cluster. (**a**) The analysis of *Dl* and *klu* expression levels in the escargot positive intestinal progenitor lineage identifies 3 populations within the *esg*^+^ ISC/EB cell cluster: *klu*^+^, *Dl*^−^ (red), *Dl*^+^, *klu*^−^ (green), and *Dl*^−^, *klu*^−^ (grey). Double positive cells are not identified confirming the mutual exclusivity of these markers. (**b**) Dot plot representation of additional lineage markers and cell cycle genes shows high expression of quiescence marker *nub* in *esg*^+^, *Dl*^−^, *klu*^−^, and *pros*^−^ quiescent ISC (qISC), whereas expression of cell cycle genes *CycE*, *CycD,* and *stg* is high in *esg*^+^, *Dl*^+^, *klu*^−^, and *pros*^−^ proliferating ISC (pISC) and expression of EB markers like *Eip75B* is highest in *esg*^+^, *Dl*^−^, *klu*^+^, and *pros*^−^ EB cell cluster. (**c**) Dot plot representation of *miR-277* target genes identify the differentially expressed FAO genes *Mtpalpha*, *yip2*, and *CG5599* as quiescent ISC (*Dl*^−^) markers. *CG3902*, *CG4860*, *CG9547*, and *whd* are higher expressed in proliferating ISC (*esg*^+^, *Dl*^+^, *klu*^−^, and *pros*^−^). *CG31075* is the only *miR-277* target gene showing higher expression in EB (*esg*^+^, *Dl*^−^, *klu*^+^, and *pros*^−^). (**d**) CG9547 protein expression levels significantly decline in the ISC lineage (*n* = 6, 8, 9; ANOVA: ** *p* < 0.01, **** *p* < 0.0001). (**e**,**f**) cartoons depicting *esg*^+^ intestinal progenitor lineage clusters can be further subdivided in qISC (grey circle), pISC (green circle) and EB (orange circle) upon expression of *Dl*, *klu*, and *pros*.

After an exhaustive search, we isolated and termed a third group, reflecting a second population of ISC that is *Dl*^−^ (‘*esg^+^, Dl*^−^*, klu^−^* and *pros^−^’*, Figure 5a,f) built on our metabolic mapping, marker gene expression, and a previous unpublished observation that under very low turnover conditions, the majority of ISC of virgin females are negative for the mitosis marker pH3 and importantly *esg^+^/Dl*^−^ [6] (Antonello and Reiff, unpublished results). We also observed that reprogramming towards lipid uptake upon mating [6] may already cause an upregulation of CG9547 protein levels in MF compared to VF (Figure S5c). Using these criteria, *Dl^+^* proliferating ISC (pISC) have high expression of cell cycle genes *CycD, CycE,* and *stg* (*string*, *CDC25A*) (Figure 5b), whereas quiescent ISC (qISC) have high levels of *nub* (*POU/OCT1*) involved in quiescence [40,41] (Figure 5b). E2F1 is also high in qISC, where it might exert its known role as repressor of OXPHOS and mitochondrial function [42,43] and promote FAO to support self-renewal and drug resistance via NANOG in tumor-initiating stem-like cells [43] (Figure 5a). EB show higher levels of endoreplication genes (*Orc1, dup*), cellular growth (*mTor, Myc*) and *Eip75B* [5] (Figure 5b).

Strikingly, qISC and pISC are characterized by high expression of the FAO genes *CG5599, Mtpalpha*, and *yip2* and, respectively, *CG3902, CG4860, CG9547,* and *whd* in comparison to EB (Figure 5c). As an exception from *miR-277* target genes showing clear expression differences among populations, *CG31075* sticks out being high in a small number of EB (Figure 5c). *CG31075* is predicted to code for an orthologue of human ALDH1A, an alcohol dehydrogenase, and is thus not involved in FAO but is part of KEGG pathway ‘Fatty Acid Metabolism’ (Figure 1b). As *CG31075* knockdown also results in reduced ISC sizes (Figure S4d,e), it is a tempting candidate for future studies involving metabolism of ISC.

These data suggest that *miR-277* target genes are differently regulated between *Dl^−^* and *Dl^+^* ISC and enriched in both populations (Figure 5c,d) compared to the cell types of the intestinal lineage. In accordance with our in silico data on ISC metabolic gene expression, this suggests that ISC are characterized by FAO and that ISC, from a metabolic point of view, are dissimilar to EB that can be clearly distinguished by published marker genes (Figure 5b–d) [5,20,44]. Together, our analysis and experiments show high expression of FAO genes in qISC gradually declining towards terminal EC differentiation (Figure 5e), a tempting lead towards understanding of ISC metabolism, which we sought to experimentally address in the following.

### 2.5. miR-277 Levels affect Midgut Homeostasis and Progenitor Survival

**Figure 6:**
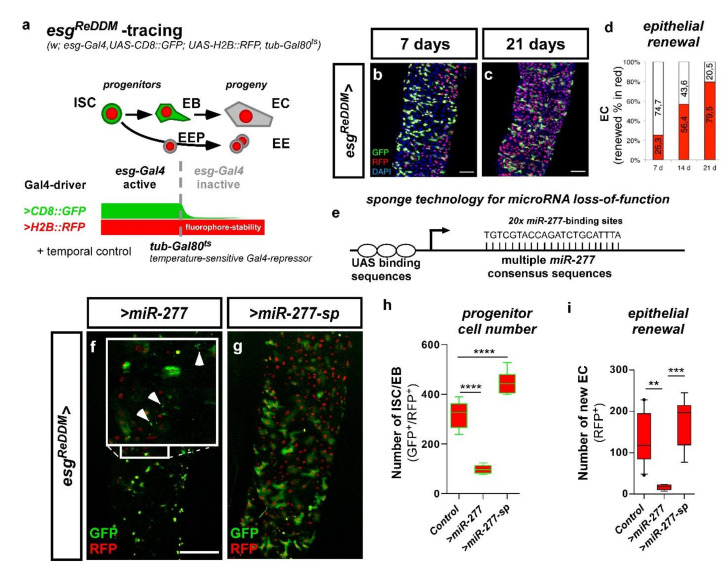
*esg^ReDDM^* tracing of stem cell production and manipulation of *miR-277* in ISC and EB. (**a**) The expression of two different fluorophores (*CD8::GFP* and *H2B::RFP*) is driven by the ISC and EB specific driver *esg-Gal4*. EB differentiating to epithelial EC loose *esg-Gal4* driven CD8::GFP, while stable H2B::RFP persists. Also, the enteroendocrine precursors (EEP) and their progeny (EE) loose the *esg-Gal4* driven CD8::GFP, but retain the H2B::RFP. The expression of UAS-driven transgenes is temporally controlled by a ubiquitously expressed temperature-sensitive Gal80^ts^ repressor, which is inactivated by a temperature shift to 29 °C. (**b**,**c**) show tracing in control (*esg^ReDDM^*>/*w^1118^*) adult *Drosophila* mated females. EB integrate in the epithelium as EC or EE (GFP^−^/RFP^+^) revealing midgut turnover rate under physiological conditions after 7 days (**b**) and 21 days at 29 °C (**c**). (**d**) Quantification of epithelial renewal in adult PMG traced for 7–21 days (**a**–**d**, modified from Antonello et al., 2015). (**e**) Schematic of loss-of-function by microRNA sponges achieved by the expression of multiple consensus sequences for the according microRNA. (**f**) *esg^ReDDM^* driven overexpression of *miR-277* resulting in cell death of presumably small GFP^+^/RFP^+^-ISC (inset arrowheads). (**g**) Depletion of *miR-277* with a UAS-driven sponge titrating intracellular *miR-277* levels. (**h**,**i**) Quantification of ISC/EB-numbers (**h**) and EC renewal (**i**) in *miR-277* overexpression and knockdown after 7 days in R5a/b (*n* = 13, 5, 8; ANOVA: ** *p* < 0.01, *** *p* < 0.001, **** *p* < 0.0001). (scale bar is 50 µm in (**b**,**c**,**f**,**g**)).

Taking advantage of the accessible and versatile genetic toolbox of *Drosophila*, we set out to investigate whether there are consequences of altered expression levels of genes involved in FAO metabolism in ISC. Like its human counterpart, the adult *Drosophila* midgut is replenished by tightly controlled ISC division and differentiation of progenitors into absorptive enterocytes (EC) and secretory enteroendocrine cells (EE) [11,12,14] (Figure 6a). To better understand the function of *miR-277* in intestinal homeostasis *,* we employed the ‘ *ReDDM’* tracing system (Repressible Dual Differential Marker) [3,21,30]. *ReDDM* allows determination of tissue turnover with temporal control of tracing and simultaneous expression of further UAS-driven transgenes. The spectrum of possible effects that we can detect and decipher using *ReDDM* ranges from proliferation, differentiation, and over apoptosis to aberrant cellular morphology [3,5,6,21,30].

The principle of *ReDDM* relies on differential marking of cells having active or inactive *Gal4* expression with fluorophores of different stability. Combined with the enhancer trap *esg-Gal4, esg^ReDDM^*, double marks qISC, pISC, and EB driving the expression of *UAS-CD8::GFP* (*>CD8::GFP*) with short half-life and *>H2B::RFP* with long half-life (Figure 6a). Crosses are grown at 18°C in which transgene expression is repressed by ubiquitous tubulin-driven temperature sensitive Gal80^ts^. By shifting adult females to 29 °C, Gal80^ts^ is destabilized, in turn enabling spatiotemporal control of *esg^ReDDM^* tracing and additional UAS-driven transgenes including *UAS-Cas9,* important in experiments making use of CRISPR-Cas9 (Figure S7a–f, *esg^ReDDMCas9^*). Upon epithelial replenishment, newly differentiated epithelial EC and EE stemming from ISC divisions retain RFP^+^-nuclei due to fluorophore stability and show gradual renewal of the intestinal epithelium (Figure 6b–d) [3].

By crossing *esg^ReDDM^* with flies carrying either UAS-constructs encoding for *miR-277* or a *miR-277-sponge (miR-277-sp)*, we investigated overexpression and knockdown of *miR-277*. MicroRNA sponges contain multiple complementary binding sites to the seed sequence of a microRNA of interest (Figure 6e) and reduce endogenous microRNA levels in both flies and human cell culture [31,32]. Intriguingly, raised levels of *miR-277* led to significantly reduced ISC and EB numbers compared to controls after seven days of tracing and transgene expression (Figure 6f,h,i). Consequently, numerical loss of ISC and EB (Figure 6h) impairs intestinal homeostasis reflected by the lack of newly generated EC (Figure 6i). Strikingly, forced *miR-277* levels affect ISC and EB survival displayed by membrane-blebbing and nuclear fragmentation (Figure 6f inset, Figure S4a), both hallmarks of apoptotic progenitors in the intestine [21].

Conversely, knockdown of *miR-277* using miR-277 sponges leads to accumulation of mature EB accompanied by low numbers of small diploid ISC (Figure 6g, morphological identification of esg^+3^). Progenitor numbers and tissue renewal is significantly increased compared to controls (Figure 6h,i), which suggests a requirement for optimal *miR-277* levels in ISC survival and differentiation of EB to epithelial EC. EE differentiation was addressed by immunostaining with the EE marker prospero [11,12,29], but is not affected by *miR-277* manipulations (data not shown).

In addition, the survival of ISC and EB upon forced *miR-277* expression is rescued by baculoviral P35 (Figure S4b,b’) underlining that the observed form of cell death is apoptosis [45]. However, rescued progenitors remain small in size and rarely divide suggestive for an additional proliferation and growth-related impact of *miR-277* (Figure S4b’). Apoptosis of ISC upon *miR-277* expression reveals their dependence on proper *miR-277* levels, which led us to hypothesize that ISC depend on proper regulation of genes involved in FAO metabolism. As microRNAs are known to post transcriptionally regulate many genes, we sought to directly address FAO with RNAi against individual FAO genes in the next experiments.

### 2.6. miR-277 and FAO Deficiency affect ISC Morphology and Subsequently Survival in Physiology and Pathology

Our findings of *miR-277-*induced apoptosis are of great interest as apoptotic mechanisms controlling ISC survival are unknown and harbor therapeutic potential as ISC are the cells of origin for colorectal cancer (CRC). Cellular growth, mitochondrial maturation and endoreplication are hallmarks during the differentiation process from ISC to EC [3,5,19,20,21,30]. In previous experiments (Figure 6f, Figure S4a) we observed that when *miR-277* induced apoptosis is blocked with p35, ISC survive although much smaller in size (Figure S4b’ arrowheads). Thus, we sought to address consequences of *miR-277*-mediated FAO gene knockdown and individual FAO gene RNAi knockdown on ISC size and survival using *esg^ReDDM^* [3,30].

**Figure 7:**
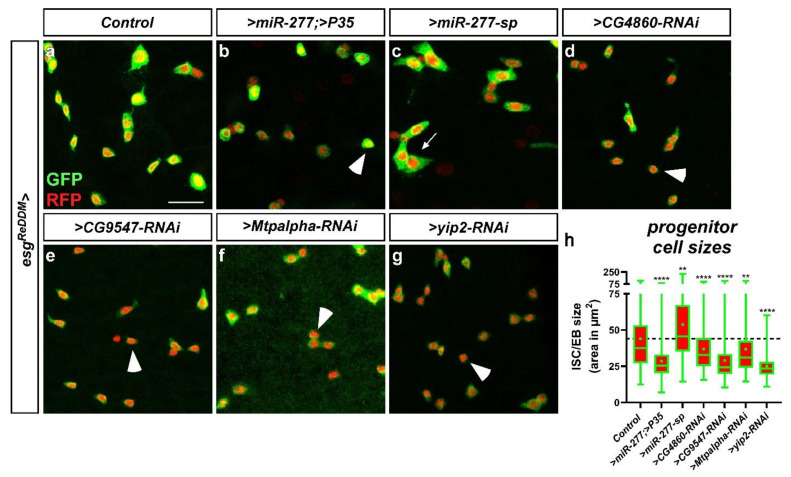
*esg^ReDDM^* tracing with manipulation of *miR-277* and *miR-277* target genes from FAO in ISC/EB. (**a**) Confocal image of control PMG (R5a/b) after 7 days showing normal ISC and EB-numbers and sizes. (**b**) Forced expression of *miR-277* with block of apoptosis by P35. Arrowheads point to small ISC, whereas arrows indicate particularly and unusually big ISC. (**c**) Knockdown of *miR-277* using *miR-277-sponges* increases the number of ISC/EB with enlarged size (**c**,**h**). (**d**–**g**) knockdown of *miR-277* target genes from FAO results in a higher number of small ISC ((**d**–**h**), arrowheads). (**h**) Quantification of ISC/EB-sizes in manipulations of *miR-277* and knockdown of *miR-277* target genes after 7 days in R5a/b (*n* = 200, 199, 150, 150, 199, 150, 150; ANOVA: ** *p* < 0.01, **** *p* < 0.0001). (scale bar is 20 µm).

After seven days of knockdown using *esg^ReDDM^,* we found no indication of apoptosis, but significantly decreased ISC size upon forced *miR-277* expression (Figure 7b) and after knockdown of individual FAO genes (*CG4860, CG9547, Mtpalpha* and *yip2*, Figure 7d–h), possibly reflecting a disruption of metabolic energy supply for metabolic growth. Conversely, knockdown of *miR-277* using *miR-277-sponges* increased ISC size (Figure 7c,h). Strikingly, *yip2,* the enzyme catalyzing the last step of FAO, also revealed the strongest impact on ISC size (Figure 7h) and followed the pseudotime differentiation (Figure 3b and Figure 5b). Together, these experiments show a strong impact of FAO genes for ISC growth and that our observation of *miR-277-*induced apoptosis is not caused by the regulation of other *miR-277* targets.

In contrast to *miR-277*, FAO gene RNAi did not result in ISC apoptosis after seven days. However, after 21 days of RNAi-mediated *CG5599* and *Mtpalpha* knockdown, we observed membrane-blebbing and nuclear fragmentation indicating apoptosis in progenitors (Figure S5j,k, arrowheads). Generally, RNAi-mediated knockdown should resemble downregulation comparable to forced expression of a microRNA. Our data in Figure S5 suggests that RNAi is probably less effective than an endogenous microRNA evolved to regulate these genes, which might account for this phenotypic delay. Together these data suggest a crucial role of FAO as energy source for ISC that is subsequently essential for their survival. Intrigued by the effects of FAO gene depletion reduction on ISC survival, we wanted to study ISC survival with greater detail in a benign pathological context.

### 2.7. miR-277 in a Benign ISC Tumor Model

The discovery of *miR-277*-mediated ISC survival is intriguing, as ISC are known to resist radiation and chemically induced apoptosis. ISC have been established as cell of origin for colorectal cancer (CRC) in the mammalian and fly intestine [46,47,48,49]. While several mitogenic factors have been described, no factors regulating ISC survival have been identified [50] and might bear high therapeutic value. We previously described that revealing cell death in vivo is challenging as dying cells are cleared off rapidly from the intestinal epithelium by macrophages [21], which hampers quantification and might result in underestimation of apoptotic cell loss.

**Figure 8:**
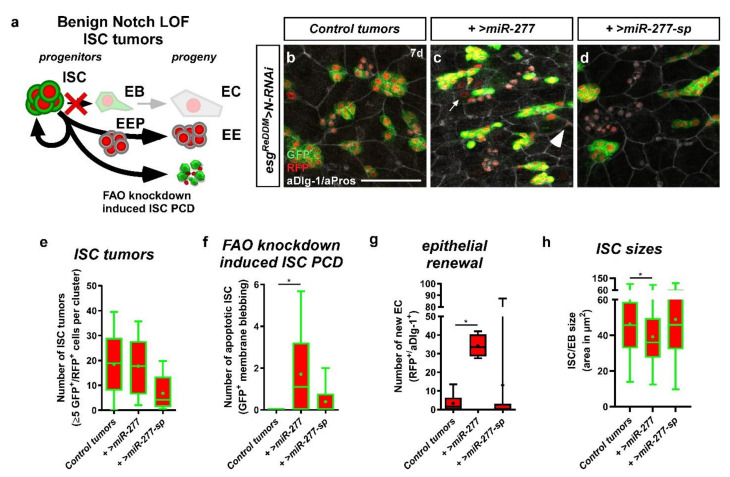
*esg^ReDDM^* tracing and manipulation of *miR-277* in the benign Notch tumor model. (**a**) N LOF in ISC/EB prevents EB specification through N signaling and EC production, thus resulting in a stochastic rate of symmetric ISC divisions (ISC tumor formation) and the production of EE (EE tumor formation). We added ISC apoptosis caused by overexpression of *miR-277* as a possible outcome for ISC division (apoptotic ISC, aCasp3 positive, Figure S6c–c’). (**b**) *esg^ReDDM^* tracing combined with *N-RNAi* driven in ISC and EB results in the formation of ISC tumors (GFP^+^/RFP^+^) and EE tumors (GFP^−^/RFP^+^/aPros^+^) and a reduced number of renewed EC (GFP^−^/RFP^+^/aDlg-1^+^) in midguts of mated female flies after 7 d at 29 °C. (**c**) Simultaneous overexpression of *miR-277* in the Notch tumor model is not affecting ISC nor EE tumor formation, but shows a significant increase in apoptotic ISC indicated by membrane blebbing (**c**,**f**, arrowhead), an increased production of newly differentiated EC (**c**,**g**, GFP^−^/RFP^+^/aDlg-1^+^, arrow), and significantly reduced ISC size (**h**). (**d**–**g**) Knockdown of *miR-277* in the Notch tumor model has no effect on tumor formation (**e**), number of apoptotic ISC (**f**), nor the number of renewed EC (**g**). (**e**–**g**) Quantification of number of ISC tumors (**e**), number of apoptotic ISC (**f**), and number of new EC (**g**) of *miR-277* manipulations in the Notch tumor model after 7 days in R5a/b normalized to an area of 100,000 µm^2^. (**h**) Quantification of ISC sizes (*n* = 8, 6, 7/8, 8, 7/8, 8, 7/75, 150, 121; ANOVA: * *p* < 0.05). (scale bar is 50 µm).

Here, we sought to circumvent this issue by raising the number of ISC using the established Notch (N) loss-of-function (LOF) tumor model (Figure 8a) [5,51]. N LOF results in the accumulation of ISC-like cells that are unable to differentiate (Figure 8a,b) [21]. Using *esg^ReDDM^* tracing in combination with N LOF and forced *miR-277* expression (Figure 8c), we found no effect on tumor number (Figure 8e), composition (Figure S6b), and size (Figure S6a). Strikingly, we found N-tumors with forced *miR-277* expression to undergo high rates of apoptotic cell death (Figure 8f and Figure S6c,c’) and ISC-like cells are significantly reduced in size (Figure 8h). In addition, new epithelial EC differentiate from the tumors, which might reflect an escape from apoptosis through differentiation (Figure 8g).

**Figure 9:**
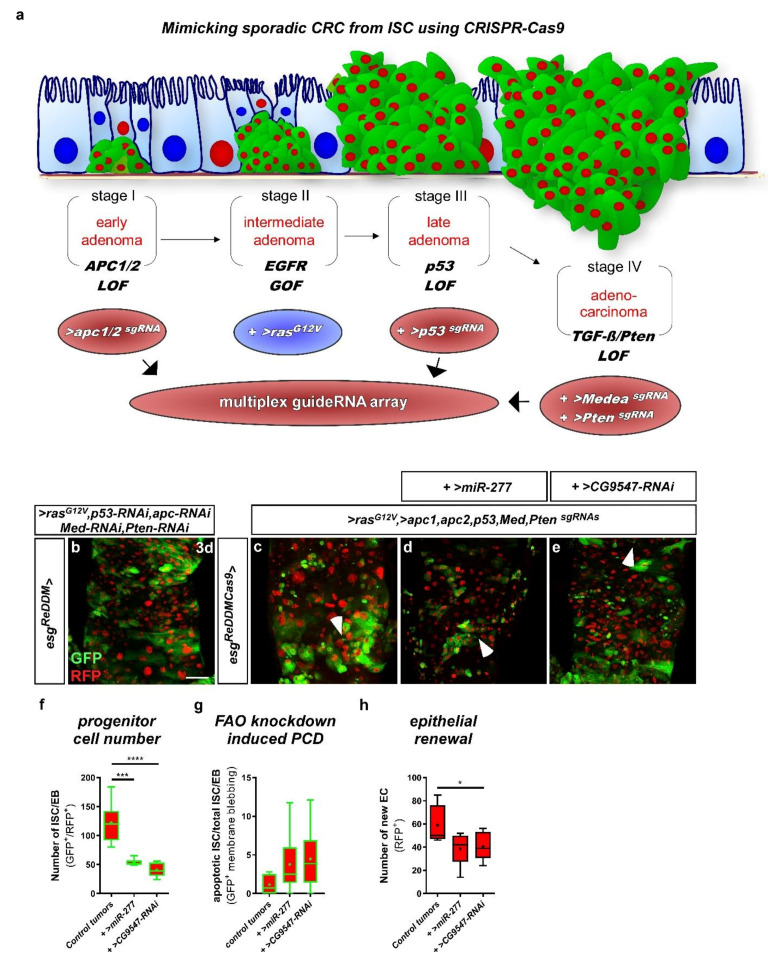
*esg^ReDDM^* tracing and overexpression of *miR-277* in a newly established colorectal cancer (CRC) model based on CRISPR-Cas9 excision. (**a**) A newly established *Drosophila* model for CRC combines the *esg^ReDDM^* tracing system with expression of *Cas9* and single guideRNAs (sgRNAs) targeting the *Drosophila* orthologs of the most frequently mutated tumor suppressors in CRC patients. Early adenoma-like lesions and hyperactive Wnt/wg signaling are induced by knockout of the Apc orthologs *apc1* and *apc2*. Additional expression of an oncogenic *ras^G12V^* leads to an activation of EGFR signaling, knockout of *p53,* and knockouts of the TGF-ß ortholog *Medea* and *Pten* mimic sporadic CRC patient-like carcinoma. In this model, the hallmarks of colorectal cancer, namely, (i) an increased SC proliferation, (ii) decreased differentiation to epithelial cells, (iii) a decreased apoptosis rate, and (iv) cell migration through the basal membrane, can be modeled and analyzed. Here, we used this model to investigate *miR-277* overexpression in CRC-like modified ISC. (**b**) *esg^ReDDM^* tracing combined with the RNAi-based *Drosophila* CRC model established by Bangi et al. 2016 results in the formation of ISC/EB cell clusters (GFP^+^/RFP^+^) and an increased EB growth in midguts of mated female flies after 7 d at 29 °C. (**c**) *esg^ReDDMCas9^* tracing combined with our new CRISPR-Cas9-induced CRC model reflects all CRC characteristics previously observed by Bangi and colleagues [49]. In detail, Cas9 excision of sporadic CRC associated genes leads to the formation of ISC/EB cell clusters, increased EB growth and PCD of ISC/EB indicated by membrane blebbing (arrowheads) in midguts of mated female flies after 3 d at 29 °C. As a consequence, overall survival of CRC flies is strongly reduced to about one week. (**d**,**e**) Simultaneous overexpression of *miR-277* (**d**) or RNAi mediated knockdown of *CG9547* (**e**) in the CRISPR-Cas9-induced CRC model reduces the number of ISC/EB compared to control tumors and reduces EB differentiation. (**f**–**h**) Quantification of ISC/EB numbers (**f**), number of apoptotic ISC/EB (**g**), and number of newly differentiated EC (**h**) of the newly established CRISPR-Cas9 induced CRC model and simultaneous overexpression of *miR-277* after 3 days in an area of 40,000 µm^2^ in R5a/b (*n* = 7,6,6/7,6,6/7,6,6; ANOVA: * *p* < 0.05, *** *p* < 0.001, **** *p* < 0.0001). (scale bar is 50 µm).

Loss of programmed cell death is observed during tumorigenesis and is a hallmark of malignant growth. The basis of the used benign model, LOF of N receptors, is not a frequent alteration observed in CRC tumorigenesis, which is why we designed and established the first *Drosophila* model of CRC that is based on CRISPR-Cas9-induced gene excision (Figure 9a and Figure S7a). Individual conditional CRISPR-Cas9 gene editing of single tumor suppressors was recently shown to induce similar benign intestinal tumors [52]. We chose to mimic sporadic CRC development by targeting frequently mutated genes simultaneously that were previously shown to induce all hallmarks of CRC [49]. For that purpose, we advanced a cloning protocol for multiplex single-guideRNA arrays involving ‘scarless’ ligation with high efficiency (Figure S9) [53].

GuideRNAs in our model are under control of a UAS promoter and target *apc1, apc2 (adenomatous polyposis coli 1&2), p53, Medea (dSmad4)* and *Pten (Phosphatase and tensin homolog)* (Figure S7g,h). Transgenic flies were injected and subsequently recombined with flies harboring oncogenic *>ras^G12V^*, reflecting EGFR signaling gain of function (Figure 9a). To allow simultaneous Cas9 excisions, tracing, and UAS-driven genetic manipulations in ISC, we recombined an *UAS-Cas9.P2* transgene into the *esg^ReDDM^* tracing flies (*esg^ReDDMCas9^*) (Figure S7a–c). In control experiments, ISC and EB appear more rounded upon Cas9.P2 expression (Figure S7c) in accordance with a previous study [52], but their numbers remain constant comparable to *esg^ReDDM^* controls (Figure S7b) and also tissue homeostasis is not disrupted after seven days (Figure S7d,e). Additionally, we approached the efficiency of CRISPR-Cas9 events and found a 99% reduction crossing single guideRNA flies targeting CD8::GFP in *esg^ReDDM^* (Figure S7f), proving highly efficient excision. One of several advantages of our CRISPR/Cas9-based model over previous CRC models [49] is the irreversibility of Cas9 excision, which also excludes possible compensation of RNAi-mediated knockdown.

### 2.8. miR-277 and Colorectal Tumorigenesis

Firstly, we compared the CRC model from Bangi et al., 2016 [49] (Figure 9b) with our new model with CRISPR-Cas9 excision of *apc1/2, p53, Medea,* and *Pten* in conjunction with oncogenic *ras^G12V^* (Figure 9a). Using *esg^ReDDMCas9^* as an ISC-specific driver for intestinal tumorigenesis [46,47,48,49], virtually all ISC should be converted into aberrant CRC tumor stem cells (Figure S7f). Intestines of both CRC models showed strong proliferation, defective differentiation, overgrowth of progenitor cells, and multilayering (Figure 9c,d) revealed by additional *esg^ReDDM^* tracing. Traced ISC and their progeny can be differentiated using specific markers for EC and EE (not shown). Progenitor cell production as well as turnover of the midgut is dramatically accelerated and after only 48–72h, the remaining hypotrophic epithelium consists of new EC only. Of note, complete renewal of the midgut epithelium in non-tumoral controls usually takes about 4 weeks [3,30].

Reprogramming of fatty acid metabolism was previously observed in various tumor entities [54]. We thus wanted to address whether *miR-277,* and thus expression levels of FAO genes, is affected in our CRC model. Therefore, we crossed the *miR-277* sensor flies to our CRC model and investigated whether *miR-277* levels change upon tumor induction (Figure S8a). *esg^ReDDM^* tracing allows distinguishing between manipulated tumoral and unmodified intestinal cells (inset Figure S8b–c’). By determining GFP fluorescence intensity, we found that GFP levels inside of tumoral tissue are significantly higher than in the surrounding cells (Figure S8d). Thus, *miR-277* levels are reduced in cells of the tumoral tissue, suggesting higher expression of FAO genes (Figure S8e).

Finally, we aimed at testing a putative favorable role of *miR-277* and FAO genes and investigated whether forced *miR-277* expression in our CRC model affects ISC-like cell survival, like in benign Notch tumors (Figure 8c,f,h). In our CRC model tumors, sporadic CRC gene deletions induce pleiotropic phenotypes including proliferation, differentiation, cellular growth, and multilayering (Figure 9a,c–e). On top of that, intestines with forced *miR-277* expression (Figure 9d) or RNAi-mediated knockdown of *CG9547* (Figure 9e), and thus, reduced levels of FAO gene expression, present further clearly distinct phenotypes (Figure 9c–e): (i) progenitor cells undergo apoptosis but with a similar frequency as controls (Figure 9g), which is probably owed to p53 deletion (Figure 9a); (ii) progenitor cell number (Figure 9f) and progeny are strongly reduced (Figure 9h).

In summary, our findings suggest an important role of *miR-277-*regulated FAO genes in intestinal stem cell metabolism. Using physiological and pathological paradigms, we show that suppressed FAO gene expression clearly affect intestinal stem cell size and once *miR-277*, in its role as a pan-FAO gene regulator, is forcedly expressed, ISC even become apoptotic, hampering tumoral growth. These findings are especially important in a pathological context as so far, no specific triggers for intestinal stem cell survival have been described. Our in vivo findings on intestinal stem cell metabolic gene expression might thus pave the way for future investigation of fatty acid oxidation as a promising new therapeutic target in colorectal cancer, but also other tumor entities relying on similar metabolic cues.

## 3. Discussion

Physiological in vivo data on stem cell metabolism is scarce owing to the complexity of experimental setup and availability of proper genetic sensors and tools. Here, we employed the fruit fly *Drosophila melanogaster,* taking advantage of its exhaustive genetic toolbox to study SC metabolism in an adult organism and investigated the metabolic gene expression profile of intestinal stem cells following leads from combined in silico resources. Building on the identification of *miR-277* as a negative regulator of fatty acid β-oxidation (FAO), our data hint to a new and quiescent ISC population that depends on lipids as an energy source. In contrast to dispensable OXPHOS [20], a previous study discovered lipolysis involved in ISC survival [55]. In functional experiments, we discovered that FAO gene expression is essential for ISC survival, providing detailed insight on the metabolic cues by identifying *miR-277* as controller of the majority of FAO enzymes, and show that they are capable of eliciting ISC starvation and subsequent apoptosis. Our data however cannot exclude effects of a *miR-277-*dependent regulation of further pathways, like BCAA catabolism [25], and further *miR-277-*regulated genes involved in similar phenotypes. ISC apoptosis can be triggered under physiological conditions, in turn disrupting tissue homeostasis, but importantly, is also observed in pathological contexts in a benign tumor model. In addition, FAO-deprived ISC show strongly reduced tumor size in a new CRISPR-Cas9 fly model of colorectal cancer.

### 3.1. A Putative Role of Fatty Acid β-Oxidation in Controlling Quiescence in Stem Cells and their Lineage

The most important cue of our study, that requires and definitely warrants future investigation, is the discovery that ISC can be metabolically subdivided into a quiescent and an active ISC population using established markers [11,12,21,29,36] and existing scRNAseq data [13]. Although our data only provide a first step towards understanding the metabolic differences between quiescent and active ISC, several of our functional investigations support this hypothesis: (i) direct FAO downregulation by *miR-277* and (ii) specific individual knockdown of FAO enzymes affects ISC size and (iii) subsequently their survival.

Stalling FAO as an energy source is thought to cause rapid depletion of acetyl-CoA [56]. Studies in yeast show that acetyl-CoA levels serve as checkpoint between quiescent and proliferative state. Furthermore, high acetyl-CoA drives the acetylation of histones with loci encoding for growth regulatory genes [57,58]. EB growth has previously been shown to depend on input from EGFR-Ras signaling and occurs in parallel with endoreplication and glycolysis during EB differentiation to polyploid absorptive EC [3,20,30,44,59]. Cellular growth is a crucial process that might require the anabolic generation of cell membrane from fatty acids. It is tempting to speculate that *miR-277* expression reflects a switching mechanism for fatty acid usage between metabolic energy generation in quiescent ISC and cell membrane generation necessary in mitotically active ISC and growing EB. In line with this, SC in the *Drosophila* testis requires mitochondria and accumulates fatty acids when mitochondrial fusion is genetically ablated [60]. Interestingly, it was previously observed that knockdown of FAO enzymes phenocopy mitochondrial fusion defects [60,61]. A single study observed mitochondria in long cellular protrusions of intestinal progenitors, but did not perform any functional studies [62]. Studying the interplay between FAO and mitochondrial function in ISC and EB will be a fascinating topic for future studies.

The prime candidate pathway switching from quiescence to active ISC state and progenitor maturation is EGFR-Ras signaling. Apart from growth, EGFR stimulates proliferation in ISC under homeostatic conditions [20,44,59,63] and deprivation of EGFR signaling leads to programmed cell death of EB [21]. Interestingly, ISC are spared from blockade of EGFR-induced apoptosis and survive as mitotically inactive singletons supported by lineage tracing in two publications [21,59]. This further supports the idea that quiescent ISC are capable of surviving without EGFR input probably by relying on FAO as an energy source. Future efforts will aim to further dissect this metabolic switch. Central to resolve this issue is to reveal specific signals and conditions driving stem cells into FAO metabolism, such as is already known for fasting [64].

### 3.2. miR-277, Fatty Acid Oxidation and ISC Apoptosis

A commonality of all investigated FAO genes is their role in mitochondrial FAO and high levels of FAO genes in ISC further support the hypothesis that ISC harbor immature mitochondria [20]. Direct molecular interactions linking apoptosis and FAO have been described in various in vitro approaches, including human CRC cell lines [65,66,67]. In accordance with our data and a role for FAO in metabolism and subsequent ISC loss, our data shows that the CPT1A orthologue, *whd* (*withered)*, diminishes progressively in the reconstructed lineages (Figure 3b). The *whd* orthologue catalyzes the transport of long-chain fatty acids from the cytoplasm to the mitochondria and *whd* mutants are highly sensitive to starvation and oxidative stress [68]. Further FAO genes follow that expression pattern from ISC declining to EC lineage: *CG3902 (acyl-CoA dehydrogenase), CG5599 (dihydrolipoamide branched chain transacylase E2), CG9547 (glutaryl-CoA dehydrogenase), Mtpalpha (mitochondrial trifunctional protein),* and *yip2 (acetyl-CoA C-acetyltransferase)* (Figure 3b and Figure 5c), which catalyzes the last step of FAO [69].

Studies in yeast support the idea of a connection between FAO and apoptosis induced at mitochondria as the *yip2* orthologue ACAA2 interacts with proapoptotic BNIP3, a known interactor of Bcl-2, controlling cell survival [69]. Furthermore, murine ISC induce FAO upon fasting which in turn improves regeneration. The same authors found that ISC diminish over time when Cpt1a, the rate-limiting carnitine palmitoyltransferase in FAO, is genetically disrupted [56,64]. In our functional experiments, we could show that FAO knockdown in ISC phenocopies *miR-277-*induced starvation and subsequent apoptosis (Figure 7a–h).

In a previous study we found that progenitors undergoing apoptosis are cleared off rapidly e, which we circumvented by genetically increasing the number of ISC with the benign Notch tumor model. FAO depletion upon forced *miR-277* expression drives a significant number of ISC into apoptosis, despite the pro-survival signal from N LOF [21]. The same experiments also showed that significantly more progenitors from N tumors escape cell death by differentiating to EC fate. This N-independent differentiation behavior was observed before for factors inducing EC fate [5,21,40]. In our case it might reflect a locally controlled metabolic switch of ISC to an early EB fate allowing OXPHOS by activating mitochondrial energy production and thus FAO independent survival and differentiation to EC.

It was also observed that mitotically active ISC in N tumors are located at the outer rim of the ISC clusters, where they are capable of receiving mitogens like the EGF-ligand *spitz* [51]. Thus, central ISC in an N tumor mass might become quiescent and, in case of additional *>miR-277,* undergo apoptosis because FAO cannot be activated (Figure 8f). Unfortunately, we and others failed to obtain reliable cleaved caspase 3 stainings in N tumors (Parthive Patel, personal communication) [51]. *miR-277-*induced apoptosis would in turn select for FAO-independent pISC, which might provide an explanation for the tendency of higher cell numbers (Figure S6a) found in our experiments. It will be an interesting topic for future studies to elucidate which factors sense and stimulate the necessity to enter quiescence.

### 3.3. The Role of miR-277 and FAO Genes in a CRC Model

Our observed dependence of ISC on FAO is of high importance as ISC are the established cells of origin for CRC in the mammalian intestine [46,47,48,49,55]. Furthermore, *Drosophila* ISC are known to resist radiation and chemically induced apoptosis [21,50], and in rodents, quiescent +4 ISC, but not LGR5^+^ ISC, are indispensable for intestinal homeostasis following radiation [70,71,72,73,74]. It is thus tempting to speculate that *Drosophila* and mouse ISC and patient CRC-SC have similar apoptotic dependencies. Until now, no other factors than FAO regulating ISC survival have been described [50,55,64]. Survival in various tumor entities depends on EGFR signaling, which is strongly altered in about two thirds of sporadic CRC patients [49]. The drugs Cetuximab and Panitumumab containing antibodies targeting EGFR signaling have become standard therapy [49,75]. Both drugs are highly effective in neoadjuvant therapy as tumor mass is strongly reduced, which facilitates timely resection before therapy resistance develops. However, resection remains the indispensable step for successful treatment as the vast reduction of tumor mass after treatments results from the reduction of transient-amplifying (TA)-like cells through (i) reduced EGFR-dependent ISC proliferation resulting in less TA cells, but more strikingly from (ii) a reduction of the more numerous, rapidly dividing TA cells. Additionally, data in *Drosophila* suggests that the analogous cell type to mammalian TA cells, the EB, respond to EGFR inhibition with apoptosis [21].

Unfortunately, fly ISC and human CRC stem cells do not share the apoptotic sensitivity of TA cells to EGFR antibody treatments, but are driven into quiescence [76]. It is tempting to speculate that quiescence and change to FAO metabolism enable CSC to survive treatment and in turn might explain rapid CRC recurrence after treatment. Drug therapy is known to exert selective pressure additionally promoting for e.g., oncogenic RAS or BRAF variants and thus contribute to the active selection for therapy-resistant tumor stem cells. Interestingly, using our newly developed CRC model, we found that introducing a mutational pattern resembling spontaneous CRC into ISC renders ISC resistant to *miR-277*/FAO-induced apoptosis. During the stepwise and stage-specific tumorigenesis, p53 mutation results in aberrant survival of tumor cells. As a consequence of loss of pro-apoptotic p53 in our CRC model, we found that ISC gain capability to withstand forced *miR-277* expression (Figure 9f) in contrary to benign Notch tumors in which apoptosis can still take place normally (Figure 8f).

Loss of p53 is a key event in human colorectal tumorigenesis and our findings may add to the understanding of metabolic changes conferring survival and possibly also further tumor properties. Indeed, data from other tumor entities shows that cancer SC utilize FAO for self-renewal and resistance to chemotherapy [43,77]. The dependence of adult stem cells on mitochondrial FAO and lipid metabolism for their maintenance is not only highlighted in our study, but has also been evidenced in mammalian hematopoietic and neural SCs [78,79]. Our data provides additional understanding underlining the dependency of ISC on FAO and its control by a microRNA. In follow up studies, the possibilities of combinatorial treatments of FAO together with targeting EGFR might be investigated and their therapeutic combined value will be evaluated to help improve future cancer treatment.

## 4. Materials and Methods

### 4.1. In Silico microRNA Target Prediction

Targets of miRNAs are usually predicted by scanning for consensus sequences of their seed sequence in mRNA. Several online tools are available that generate lists of genes predicted to be regulated by a given miRNA. Depending on the stringency of the algorithm used in a particular prediction tool, dozens, up to the magnitude of thousands, of genes are predicted to be targets of a particular microRNA. To combine lists from four different prediction tools (Miranda, Pictar, Targetscan and a miRNA target collection from Bielefeld), we extracted multiple hits from all four tools and restricted the final list of eight genes that showed up in at least three of four predicted target lists (see Figure 1). The list of investigated microRNAs encompasses *miRs-7, 8, 14, 34, 124, 277, 278, 315.*

An earlier in silico study on *miR-277* also identified the BCAA degradation pathway. However, it did not identify multiple hits in endocytosis nor fatty acid metabolism like our study, probably due to the early developmental state of prediction tools for miRNA targets [25,80]. As the vast majority of genes stemmed from FAO, our study focused on FAO metabolism and the role of *miR-277* in ISC.

### 4.2. Genetics and fly Husbandry/Fly Strains

The following transgenic flies were employed: *esg^ReDDM^* [3], *NRE::mCherry* [81], *Gbe+Su(H)dsRed* (T. Klein), *UAS-miR-277* [25], *Mex-Gal4*, *UAS-P35* (Bruce A. Hay), *UAS-ras^G12V^,UAS-p53-RNAi,UAS-apc-RNAi;UAS-Med-RNAi,UAS-Pten-RNAi* [49]. *Ubi-GFP* and *Ubi-miR-277::GFP* sensor flies are kindly provided by Klaus Förstemann. From Bloomington Drosophila Stock Center (BDSC): *UAS-miR-277-sponge* (BL61408), *UAS-CG4860-RNAi* (BL67769), *UAS-CG9547-RNAi* (BL53327), UAS-Mtpα-RNAi (BL32873), *UAS-yip2-RNAi* (BL36874), *UAS-CG4389-RNAi* (BL32873), *UAS-CG5599-RNAi* (BL32876), *UAS-Cas9.P2* (BL58985), *vas-phiC31;attP51C* (BL24482), *vas-phiC31;;attP86Fb* (BL24749), *UAS-ras^G12V^* (II) (BL64195), *UAS-ras^G12V^ (III)* (BL64196), *U6-EGFP-sgRNA* (BL79393), *UAS-CG31075-RNAi* (BL50654), *UAS-mCherrymitoOMM* (BL66532). From Vienna Drosophila Resource Center (VDRC): *UAS-N-RNAi* (GD14477), *CG9547::sGFP* (v318106).

### 4.3. Food Composition and Fly Keeping

Fly food contained 1424 g corn meal, 900 g malt extract, 800 g sugar beet syrup, 336 g dried yeast, 190 g soy fluor, 100 g agarose, 90 mL propionic acid, and 30 g NIPAGIN powder (antimycotic agent) in 20 L H_2_O. Food was cooked for about an hour to reduce bioburden, then filled into small plastic vials and cooled down to RT. Flies were kept at 25 °C except for crosses with temperature-sensitive GAL80ts (GAL4 repressor) which were kept at 18 °C (permissive temperature) until shifted to 29 °C (restrictive temperature) to activate GAL4-mediated transgene expression. Crosses with *esg^ReDDM^* were carried out as described previously [3,6,30]. Due to persisting problems with mucous formation on food surface in vials with VF, all experiments distinguishing between mated and virgin female flies were run on food with twice the amount of NIPAGIN. Mucous formation was avoided because of massive induction of tissue renewal by pathogenic stress.

### 4.4. RNA Isolation and cDNA Synthesis

The midguts from at least 5 mated female flies were dissected and transferred into a droplet of RNA *later* Solution (Invitrogen by Thermo Fisher Scientific, Bremen, Germany) on ice. The dissected tissue was homogenized in 100 µL peqGOLD TriFast (VWR Life Science) and total RNA was isolated as specified by the manufacturer. The following cDNA synthesis was performed with 250 ng of total RNA and the SuperScript IV Reverse Transcriptase (Invitrogen by Thermo Fisher Scientific) using a 1:1 mixture of oligo-dT primers and random hexamers directly upon RNA isolation. Prior to Real-time qPCR, cDNA samples were diluted 1:4 in dH_2_O.

### 4.5. Real-Time qPCR and Conventional PCR

Expression levels of predicted *miR-277* target genes were determined upon forced expression of *miR-277* in enterocytes of midguts from adult *Drosophila*. *Mex^ts^>* flies were crossed to *w^1118^* (control) or *>miR-277* flies at 18 °C and their progeny shifted to 29 °C for 24 h prior to RNA isolation and cDNA synthesis before running the qPCRs. After an enzyme activation step (2 min 95 °C), 40 cycles of denaturation (15 s 95 °C), primer annealing (20 s 58 °C) and elongation (30 s 72 °C) were run. SYBR Green intensities were measured at the end of every elongation step and a melting curve was calculated at the end of the PCR reaction. Primers were designed to anneal at 59 °C. Reaction was set up with KAPA SYBR FAST Universal (Roche) in a total volume of 10 µL. All qPCR results were normalized to the house-keeping gene *rp49*. For gel visualization (Figure 1c), the qPCR protocol was used and visualized on 2% agarose gel (Table 1).

### 4.6. Plasmid Cloning

For cloning of multiple sgRNAs into the pCFD6 plasmid [82], a SapI restriction site in pCFD6 was removed by site-directed mutagenesis (SDM) using a pfu polymerase (Promega) following the QuikChange II-E-Site-Directed Mutagenesis Kit Manual (Agilent). The following primers were used for SDM: pCFD6_SDM_noSapI_for TTGCGTATTGGGCGCACTTCCGCTTCC and pCFD6_SDM_noSapI_rev GGAAGCGGAAGTGCGCCCAATACGCAA to generate the pCFD6_noSapI plasmid. Successful removal of the SapI restriction site was verified by Sanger Sequencing.

### 4.7. gRNA Design

The CRISPR Optimal Target Finder [83] was used to design gRNAs and to search for possible off-targets. Gene regions of the tumor suppressors *apc1, apc2, p53, Med, and Pten,* which are orthologous to the most frequently mutated genes in CRC patients [49], were copied into the target finder. Additionally, a gRNA targeting the EGFR signaling repressor *capicua (cic)* was designed to induce an activation of EGFR signaling, which is the second most common step in colon carcinogenesis. CRISPR targets with a length of 20 bp and a 5′ NGG were identified using *Drosophila melanogaster* (reference genome, r_6) as reference. The specificities of identified CRISPR targets were evaluated using the CRISPR Optimal Target Finder [83] with maximum stringency searching for NGG and NAG PAMs in the *Drosophila melanogaster* (reference genome, r_6) reference genome. For each gene the CRISPR target with the lowest number of off-targets was selected (Table 2).

Forward primers for gRNA amplification were designed containing a BbsI or SapI restriction site, the selected guide sequence, and a sequencecomplementary to the gRNAcore on the pCFD6_noSapI plasmid (5′-3′). Reverse primers for gRNA amplification were designed containing a BbsI or SapI restriction site matching the forward primer, two SapI or BbsI restriction sites contrariwise to the flanking restriction sites, and a sequence complementary to the tRNA sequence of the pCFD6_noSapI plasmid (5′-3′). A linker between the flanking BbsI or SapI restriction sites and the remaining primer sequence was added to enable seamless cloning of multiple gRNAs. The last guide sequence targeting Pten was added to a final reverse primer, which lacks additional restriction sites. PCR reactions were performed using the Q5^®^ High-Fidelity DNA Polymerase (NEB, New England Biolabs) and the pCFD6_noSapI as template DNA. Resulting in fragments of gRNA, gRNAcore, tRNA and two SapI or BbsI restriction sites flanked by BbsI or SapI restriction sites (Figure S9).

The pCFD6_noSapI plasmid contains a gRNAcore followed by a tRNA sequence which are flanked by BbsI restriction sites. The DNA fragment containing the first gRNA1 followed by the gRNAcore sequence, the tRNA sequence, and two SapI restriction sites is also flanked by BbsI restriction sites. This fragment is combined with the plasmid by cutting with the BbsI restriction enzyme prior to ligation. The next DNA fragment contains the second target sequence, gRNA2, and two BbsI restriction sites. This fragment is flanked by SapI restriction sites and added to the plasmid by cutting with the SapI restriction enzyme and ligation. The third DNA fragment is designed as the first one with except for the gRNA sequence, along with others. To create these fragments, primer pairs were designed with forward primers binding the gRNAcore sequence on the plasmid and the specific gRNA sequence and flanking restriction site in a primer extension. The reverse primers consist of a sequence complementary to the tRNA sequence in the plasmid and the specific restriction sites in an extension (Table 3).

The pCFD6_noSapI plasmid and the DNA fragment containing the first gRNA flanked by BbsI restriction sites were cut by the BbsI restriction enzyme (NEB, New England Biolabs) prior to ligation of the fragment into the plasmid using a T4 ligase (NEB, New England Biolabs). In the next step, the created plasmid now containing the two SapI restriction sites 3′ of the first gRNA and the second DNA fragment containing the next gRNA flanked by SapI restriction sites were cut by the SapI restriction enzyme (NEB, New England Biolabs) prior to ligation. These steps were repeated until the gRNAs of all six genes were ligated into the pCFD6_noSapI plasmid. The resulting construct was injected into flies containing a phiC31 integrase and an attP docking site.

Transformant flies carrying the construct were identified by eye color produced by a mini-white gene which is inserted into the construct. These flies were used to establish stocks in single crosses. Later successful excisions mediated by the gRNA construct and the Cas9 protein were verified by PCR using primers flanking the targeted gene regions and genomic DNA from fly guts of *esg^ReDDMCas9^* controls and *esg^ReDDMCas9^>apc1,apc2,cic,Med,p53,Pten ^sgRNAs^* as templates. A knockout of *cic* could not be verified; instead, the *UAS-ras^G12V^* transgene was added to activate EGFR signaling and the *cic-sgRNA* was left out in the genotypic description.

### 4.8. Immunohistochemistry

Dissected guts from mated female flies were fixed in 4% PFA in 1XPBS for 45 min. After fixation the guts were washed with 1XPBS for 10 min and stained with primary antibodies 1:500 anti-Ssk (rabbit; [84]); 1:250 anti-Dlg-1 [mouse; Developmental studies Hybridoma Bank (DSHB)]; 1:250 anti-Pros [mouse; Developmental studies Hybridoma Bank (DSHB)], 1:200 anti-Casp3 [rabbit, Cell signalling technology cleaved caspase-3 (Asp175)], 1:200 anti-GFP [chicken, Abcam (ab13970)], 1:200 anti-Dl [mouse, Developmental studies Hybridoma Bank (DSHB, C594.9B)]) diluted in 0.5% PBT (0.5% Triton (Sigma-Aldrich) in 1XPBS) + 5% normal goat serum (Thermo Fisher Scientific, Berman, Germany). Primary antibody staining was performed at 4 °C over night on an orbital shaker. Next, guts were washed with 1XPBS for 10 min and incubated with secondary antibodies (1:500 Goat anti-RabbitAlexa568 [Invitrogen], 1:500 Goat anti-MouseAlexa647 [Invitrogen], 1:500 Goat anti-RabbitAlexa647 [Invitrogen], 1:500 Goat anti-chickenAlexa488 [Invitrogen]) and DAPI (1:1000; 100 µg/mL stock solution in 0.18 M Tris pH 7.4; DAPI No. 18860, Serva, Heidelberg) for at least 1.5 h at RT. After washing with 1XPBS for 10 min the stained guts were mounted in Fluoromount-G Mounting Medium (Electron Microscopy Sciences).

### 4.9. Image Acquisition

The posterior parts of stained midguts were imaged using a LSM 710 confocal microscope (Carl Zeiss) using ‘Plan-Apochromat 20 × /0.8 M27′ and ‘C-Apochromat 40 × /1.20 W Corr M27′ objectives. Image resolution was set to at least 2048 × 2048 pixels. Focal planes with 1 µm distance were scanned and combined into Z-stacks to image one cell layer of the tubular gut and to compensate for gut curvature.

### 4.10. Quantification of Proliferation, Cell Size and Fluorophore Intensity Measurements

Quantification of progenitor cell number and epithelial renewal and fluorescence intensity measurements were performed as described previously [5]. Fiji (ImageJ 1.51 n, Wayne Rasband, National Institutes of Health, USA) was used to calculate maximum intensity images from z-stack images. GFP positive progenitor cells of *esg^ReDDM^* [3] guts were counted manually whereas RFP positive renewed epithelial cells were counted semi-automatically by a self-written macro for Fiji. Cell size measurements were performed in Fiji by outlining the single cells by hand and measuring the area.

Midguts of *Ubi-miR-277::GFP* sensor flies and the *Ubi-GFP* controls or *CG9547::sGFP* flies were scanned with fixed laser settings and exposure times. Mean intensities of manually selected areas were determined using Fiji.

### 4.11. Statistical Analysis

GraphPad Prism 9.0.0 was used to run statistical analysis and create graphs of quantifications. For single comparisons, data sets were analyzed by two-sided unpaired *t*-test. Multiple comparisons were analyzed by one-way ANOVA and Turkey’s post-hoc test. Significant differences are displayed as * for *p* ≤ 0.05, ** for *p* ≤ 0.01, *** for *p* ≤ 0.001 and **** for *p* ≤ 0.0001.

### 4.12. Metabolic Landscape of Adult Drosophila Midgut at Single Cell Level

#### 4.12.1. Preprocessing

Previously published single-cell RNA sequencing data derived from 10,605 midgut epithelial cells from 7-d-old females were retrieved from Gene Expression Omnibus accession GSE120537 [13]. Gene expression values were gene length normalized in TPM (transcripts per million) space and log_2_ transformed. For genes associated with multiple transcripts, the longest transcript length was used. Transcript lengths were obtained from Ensembl Biomart (https://m.ensembl.org/index.html, accessed on 5 November 2021). Missing gene expression values were input using scImpute algorithm with default settings and only applied to genes with dropout rates larger than 50% to prevent over-input [85]. Metabolic gene lists were downloaded from KEGG database (http://www.kegg.jp/, accessed on 5 November 2021). Input expression values were used for t-SNE clustering [86] using Rtsne package with default settings after Krijthe, J. H. Rtsne: T-Distributed Stochastic Neighbor Embedding using Barnes-Hut Implementation. https://github.com/jkrijthe/Rtsne, 2015, accessed on 5 November 2021].

#### 4.12.2. Normalization

Four normalization methods were evaluated. Upper-quartile [87] and trimmed mean of M-values [88] were implemented using calcNormFactors function from the edgeR package [88]. Relative log expression [89] was implemented from DESeq2 [90], Deconvolution normalization using the scran package computed tumor subgroup-specific size factors [91]. Read counts were divided by size factors corresponding to tumor subgroup and then transformed back to TPM. Only genes with dropout rate <0.75 were used as reference genes for normalization to avoid noise from low-expressed genes. Performance of the methods was evaluated using the distributions of relative gene expression values amongst different cell types. The deconvolution normalization derived expression values were used, as it was most effective in minimizing the differences in the distributions of relative gene expression levels between the cell types (Figure S2c).

#### 4.12.3. Pathway Activity Analysis

The pathway score analysis from Xiao et al. was used with the input and deconvolution-normalized values [92]. The pathway activity score is defined as the relative gene expression values averaged over all genes in a specific pathway and all subgroup cells of this type [92]. Statistical significance of pathway activities in specific subgroups was calculated by random permutation test, where subgroup labels were randomly shuffled 5000 times to simulate null distribution, followed by comparison of pathway activity scores to original scores.

All code is available from the authors upon request.

## Figures and Tables

**Table 1:** List of primers used in real-time qPCR to investigate expression levels of predicted target genes of *miR-277*.

Primer	Forward (5′-3′)	Reverse (5′-3′)
*Rp49*	TGGTTTCCGGCAAGCTTCAA	TGTTGTCGATACCCTTGGGC
*miR-277*	GCGTGTCAGGAGTGCATTTG	GATTGTACGTTCTGGAATGTCGT
*CG31075*	TCCGAGGGAGATAAGGCTGA	GAATGCCTTGTCCCGATCCA
*CG3902*	CTTCTCCCTGAAGACCGTCG	GGATGGCTACCGTGGCATTA
*CG4860*	CGACCGGGAGGAGCTTTATC	TCCAATCCGGAACCACCATAC
*CG5599*	TCGATGACGGAATCCCTGAAAA	TCTCCTTGGCCACTAACTGC
*CG9547*	CAAGCTGATTGGTGCCTTTGG	GCGCACTAGTAATCCACGTCT
*MtpAlpha*	CCAGTCCTTCGTCATGGACA	CACGGATCACATCGAGAATCTTCA
*whd*	AACTTCTACGGCACGGATGC	TGCCCTGAACCATGATAGGC
*Yip2*	CATGAGTTGCAGCGCAAGAAG	GCTGTAGGATTAGACAGCCTCG

**Table 2:** List of genes and the specific sequence targeted in the CRC model with number of off-targets predicted by the CRISPR Optimal Target Finder [83].

Targeted Gene	Target Sequence	Number of Off-Targets
*apc1*	GGGCATCGCCGAGCTCAGTC	3
*apc2*	GGAGAGACGATCCGCTCAGA	5
*cic*	GGCTTGCCCGGGGAGCTTAG	4
*p53*	GGCTATTACGTGCCCCAATA	5
*Med*	GGTGAAGGACGAATACTCAG	1
*Pten*	GACGGTTTCTGAATAGGCCC	4

**Table 3:** List of Primers used to amplify gRNA constructs for the CRC model (Colors recapitulate Figure S9).

Primer	Sequence (5’-3’)
BbsI_apc1_for	ATAAGAAGACCTTGCAGGGCATCGCCGAGCTCAGTCGTTTCAGAGCTATGCTGGAAAC
SapI_apc2_for	ATAAGCTCTTCCTGCAGGAGAGACGATCCGCTCAGAGTTTCAGAGCTATGCTGGAAAC
BbsI_cic_for	ATAAGAAGACCTTGCAGGCTTGCCCGGGGAGCTTAGGTTTCAGAGCTATGCTGGAAAC
SapI_p53_for	ATAAGCTCTTCCTGCAGGCTATTACGTGCCCCAATAGTTTCAGAGCTATGCTGGAAAC
BbsI_Med_for	ATAAGAAGACCTTGCAGGTGAAGGACGAATACTCAGGTTTCAGAGCTATGCTGGAAAC
final_rev_Pten_BbsI	ATAAGAAGACCCAAACGACGGTTTCTGAATAGGCCCTGCACCAGCCGGGAATCGAACC
universal_rev_2xSapI_BbsI	ATAAGAAGACCCAAACTGAAGAGCTGAACGGCTCTTCTGCACCAGCCGGGAATCGAACC
universal_rev_2xBbsI_SapI	ATAAGCTCTTCAAACTGGTCTTCTGAAGGGAAGACTATGCACCAGCCGGGAATCGAACC

## Data Availability

The data presented in this study are available in the article and supplementary materials.

## Supplementary Materials

The following supporting information can be downloaded at: https://www.mdpi.com/article/10.3390/metabo12040315/s1. Figure S1: Overview of metabolic pathways in biological systems, Figure S2: Analysis of metabolic pathways and their activity in different cell types, Figure S3: Subclustered quiescent ISC (esg^+^, Dl^−^ and klu^−^) within the metabolic ISC cluster are negative for expression of the EE marker pros, Figure S4: esgReDDM tracing of ISC/EB with forced miR-277 expression and block of apoptosis, Figure S5: esgReDDM tracing and manipulation of miR-277 target genes CG4389 and CG5599 for 21 days and endogenous CG9547 expression in different cell types, Figure S6: esgReDDM tracing and manipulation of miR-277 in the Notch tumor model, Figure S7: Comparison of the esgReDDM and esgReDDMCas9 system and schematic composition of the newly developed CRISPR-Cas9 CRC model, Figure S8: miR-277-expression in CRC tumors revealed by miR-277::GFP sensor flies, Figure S9: schematic illustration of the strategy for seamless cloning of multiple gRNAs into the pCFD_noSapI plasmid.
