# Supplementary figures and images for: The MicroRNA miR-277 Controls Physiology and Pathology of the Adult Drosophila Midgut by Regulating the Expression of Fatty Acid β-Oxidation-Related Genes in Intestinal Stem Cells
Source: Metabolites. 2022 Mar 31;12(4):315. doi: 10.3390/metabo12040315 (PMC9028014; doi:10.3390/metabo12040315)

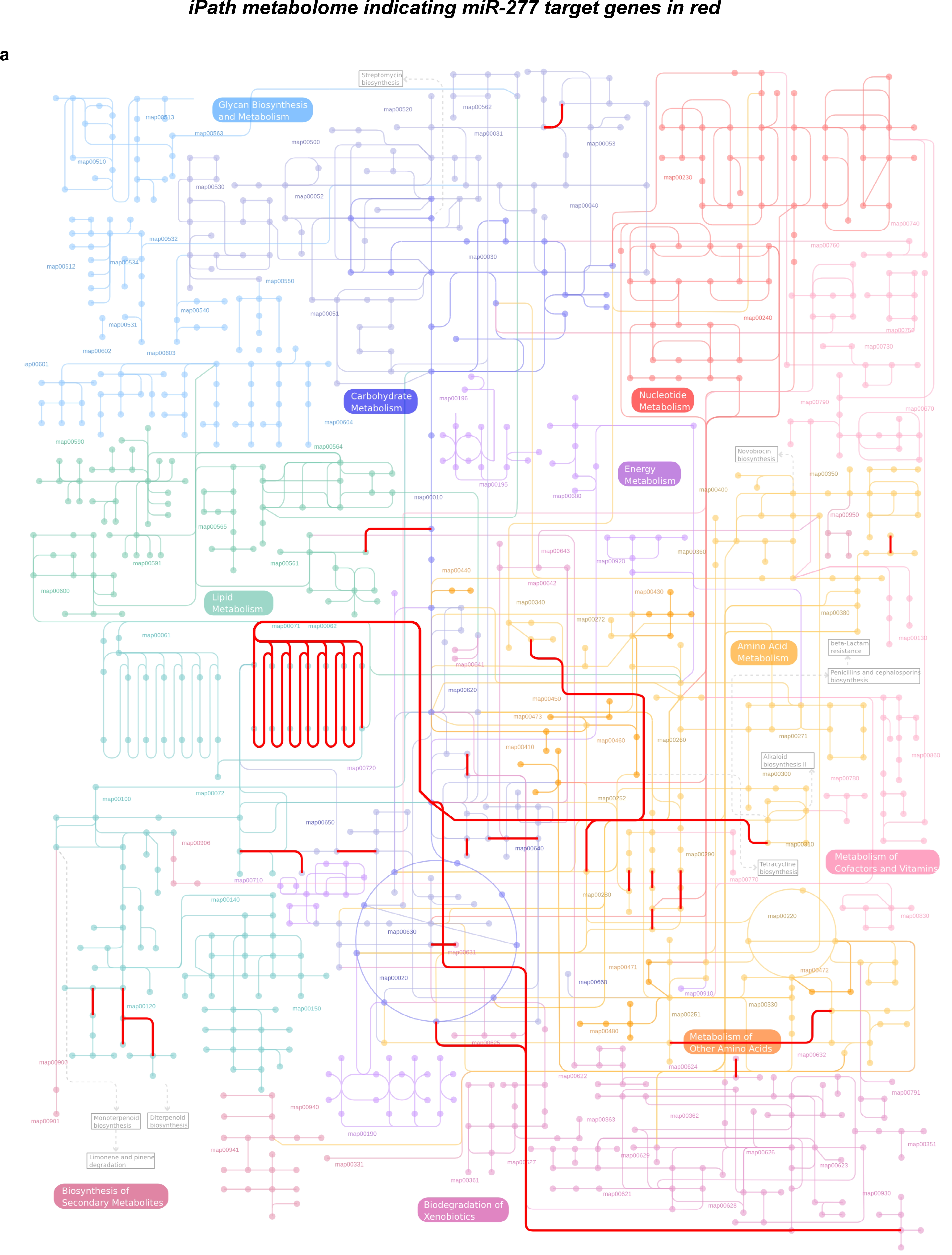

Supplement: Supplementary file 1 [file metabolites-12-00315-s001.zip › metabolites-1660805-supplementary/Supplementary Figure S1.jpg]

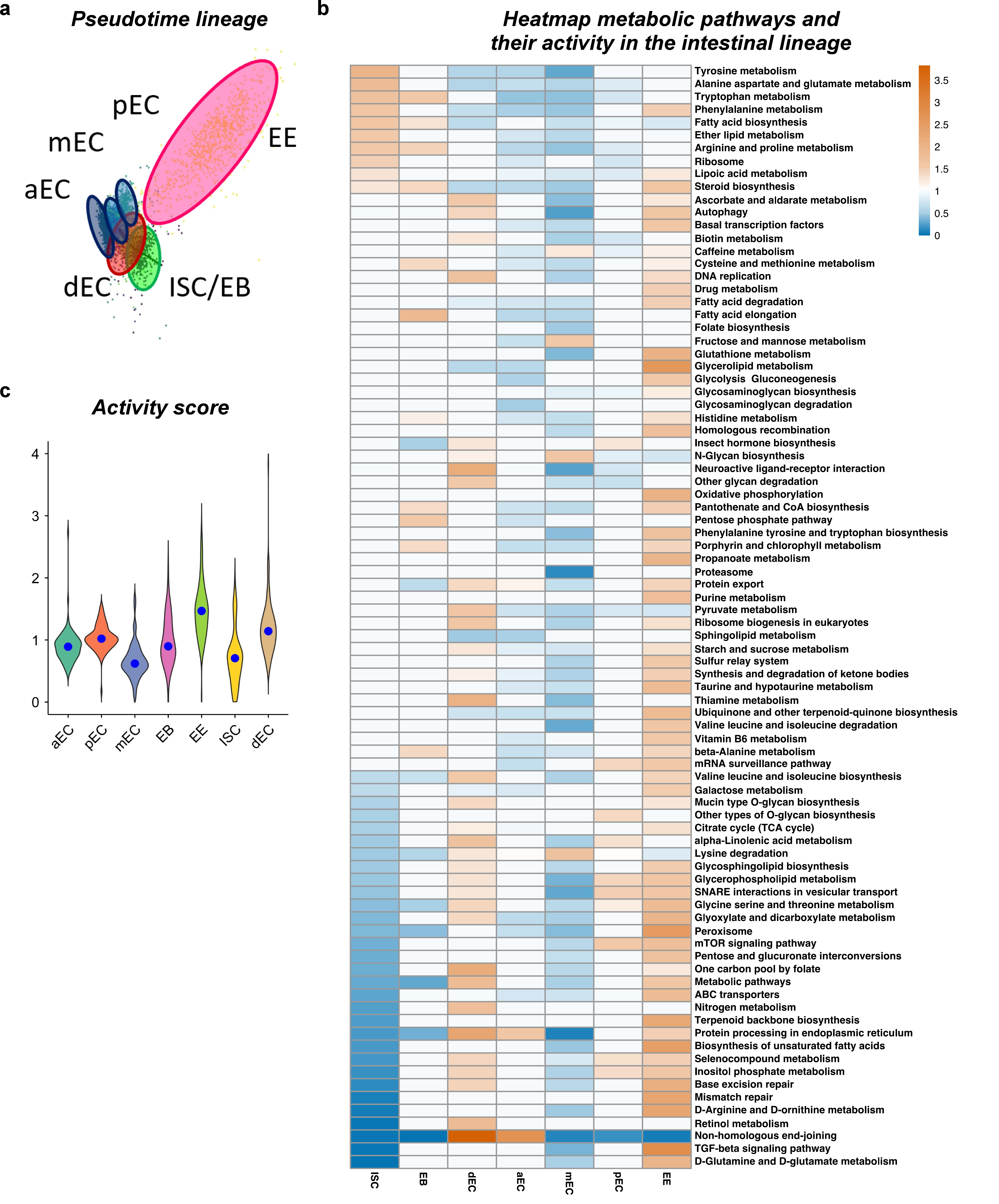

Supplement: Supplementary file 1 [file metabolites-12-00315-s001.zip › metabolites-1660805-supplementary/Supplementary Figure S2.jpg]

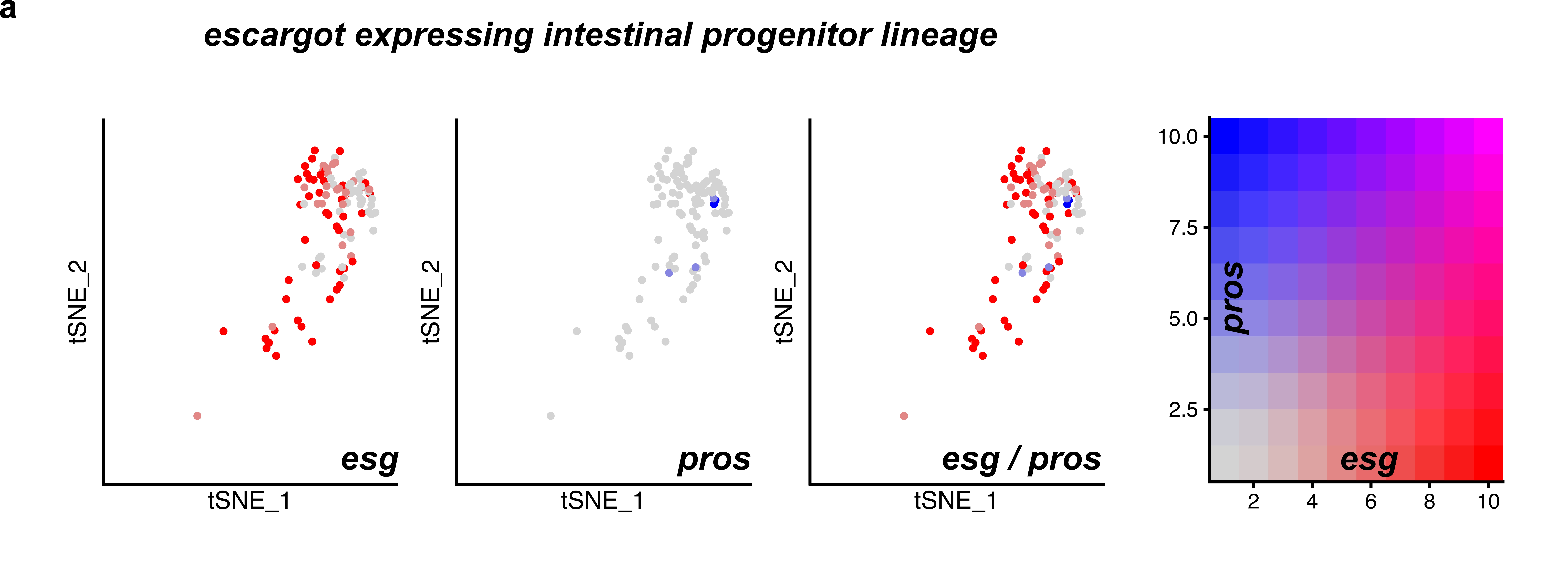

Supplement: Supplementary file 1 [file metabolites-12-00315-s001.zip › metabolites-1660805-supplementary/Supplementary Figure S3.jpg]

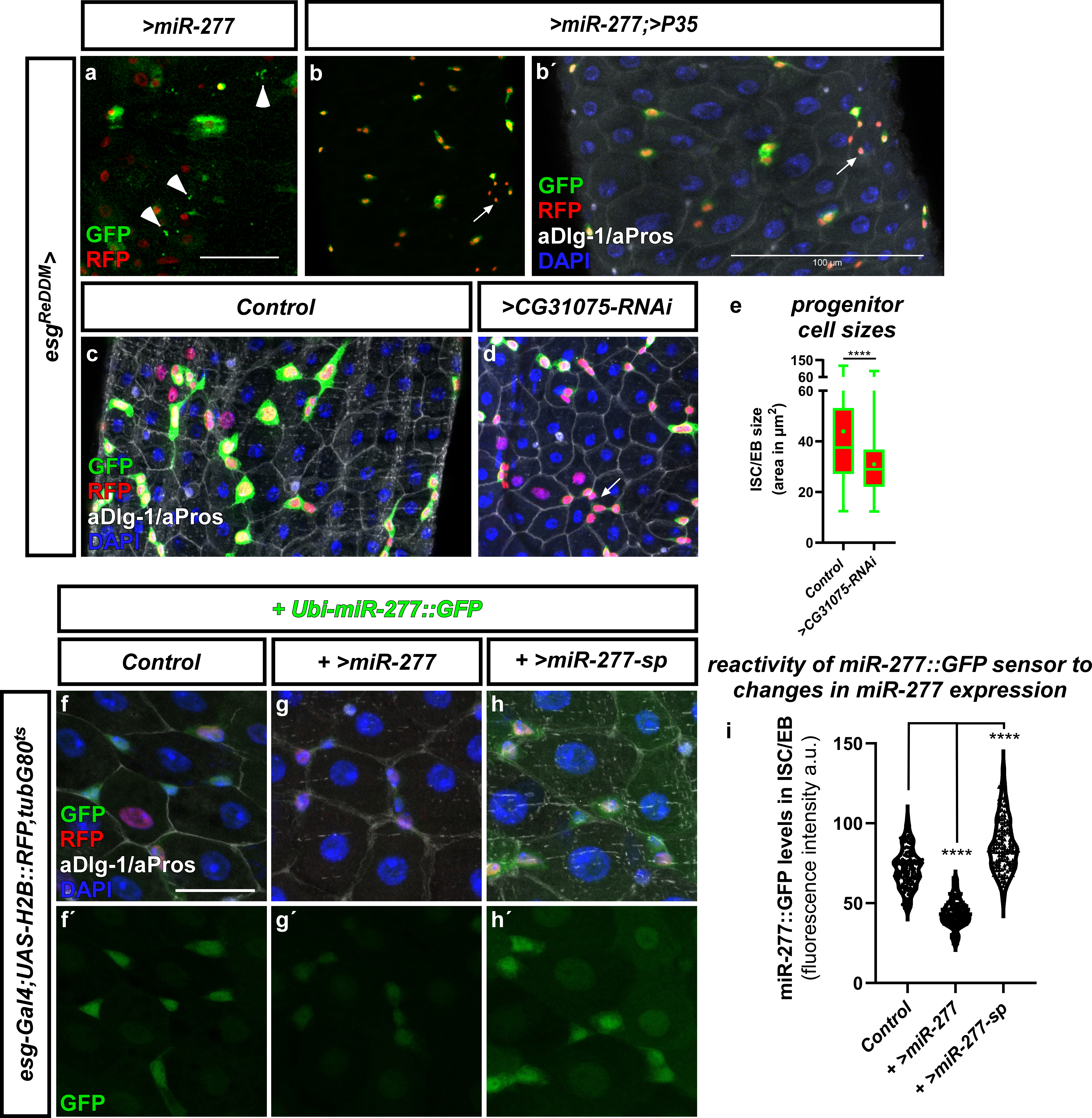

Supplement: Supplementary file 1 [file metabolites-12-00315-s001.zip › metabolites-1660805-supplementary/Supplementary Figure S4.jpg]

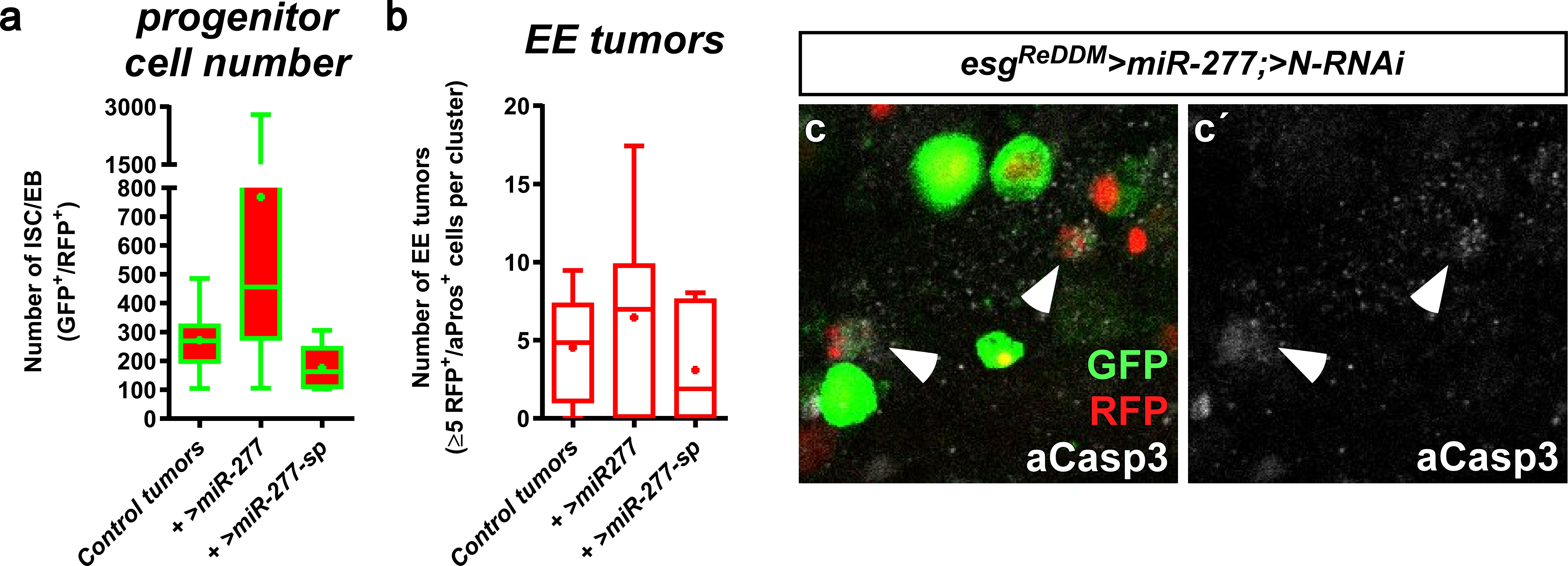

Supplement: Supplementary file 1 [file metabolites-12-00315-s001.zip › metabolites-1660805-supplementary/Supplementary Figure S6.jpg]

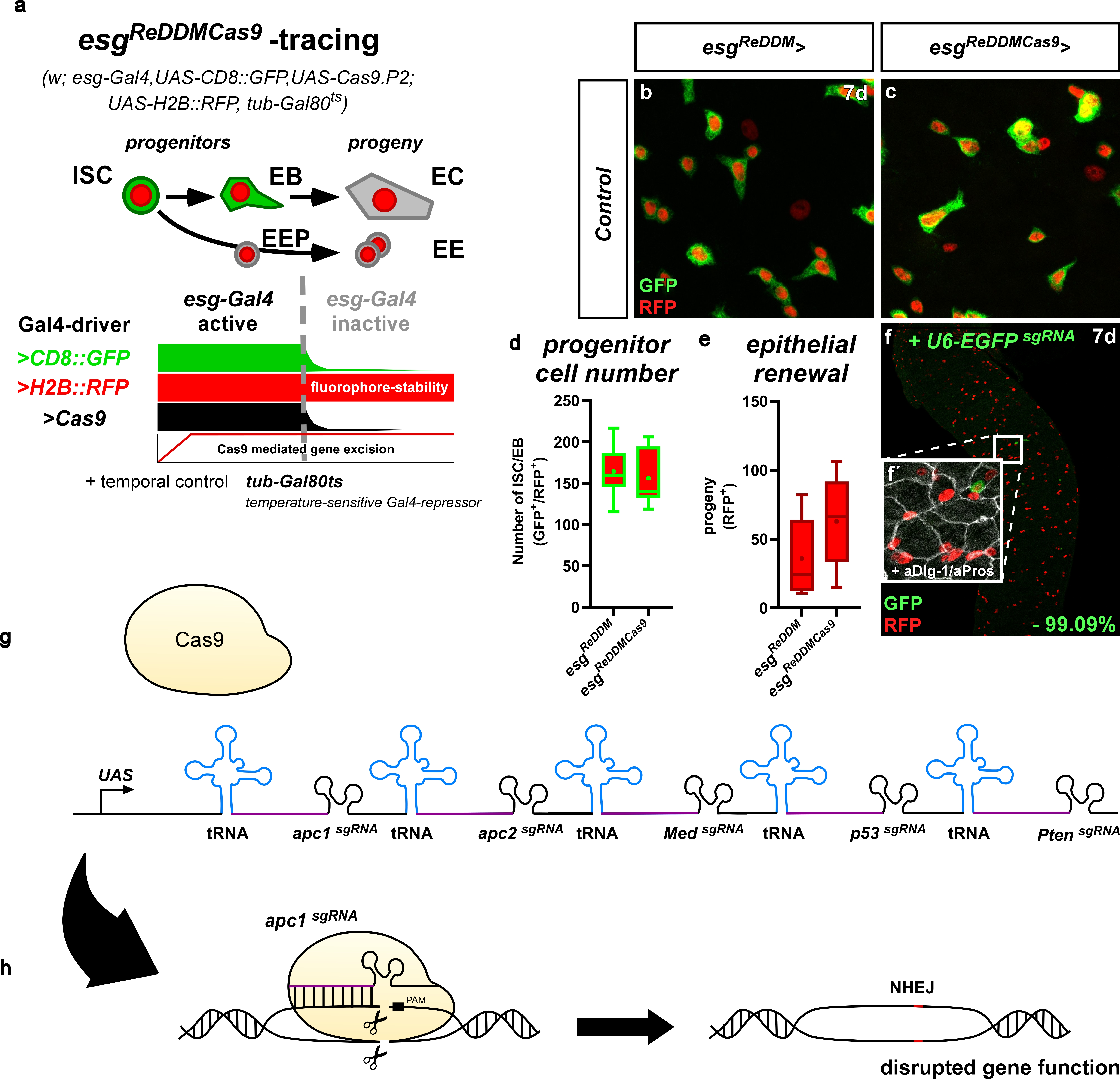

Supplement: Supplementary file 1 [file metabolites-12-00315-s001.zip › metabolites-1660805-supplementary/Supplementary Figure S7.jpg]

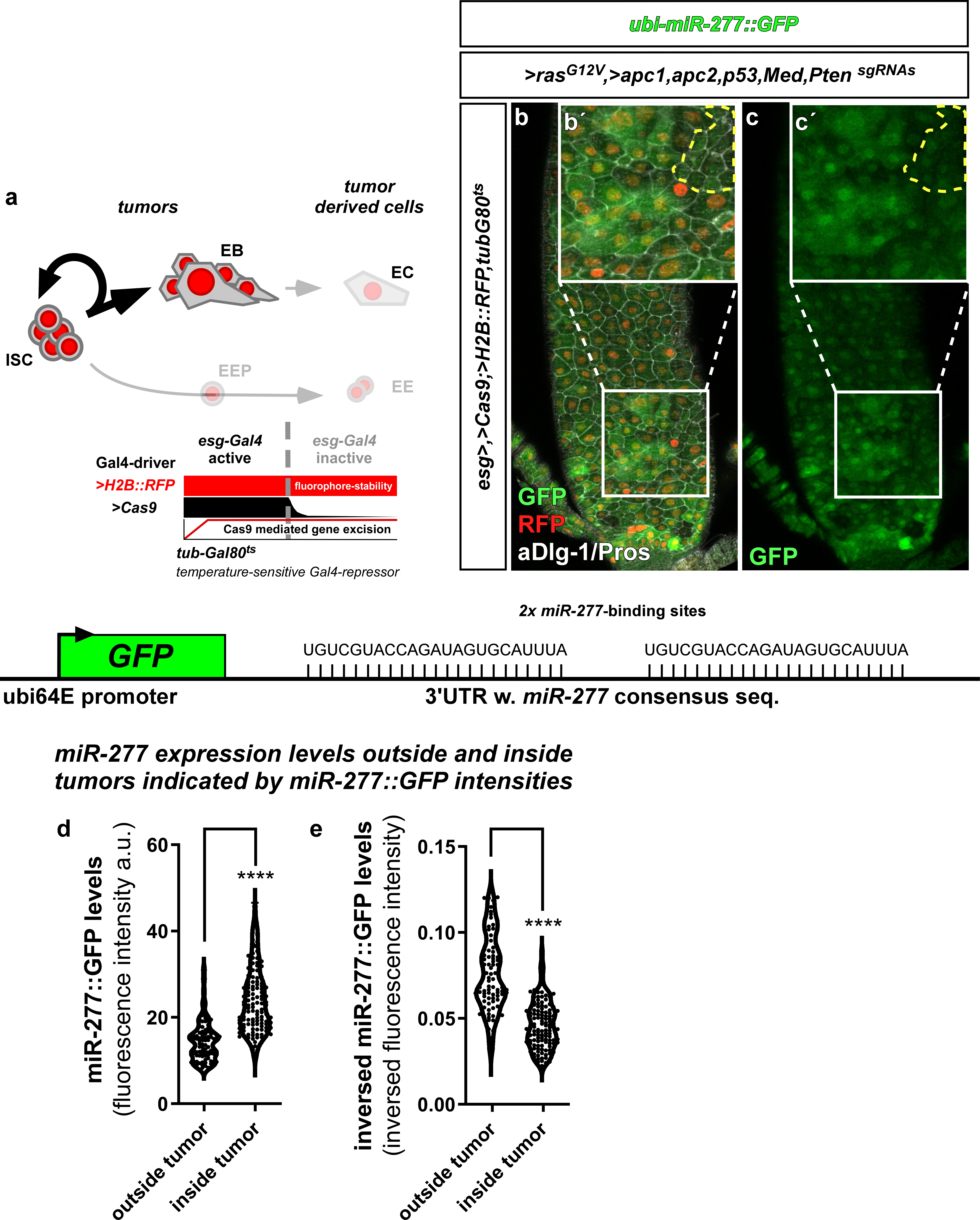

Supplement: Supplementary file 1 [file metabolites-12-00315-s001.zip › metabolites-1660805-supplementary/Supplementary Figure S8.jpg]

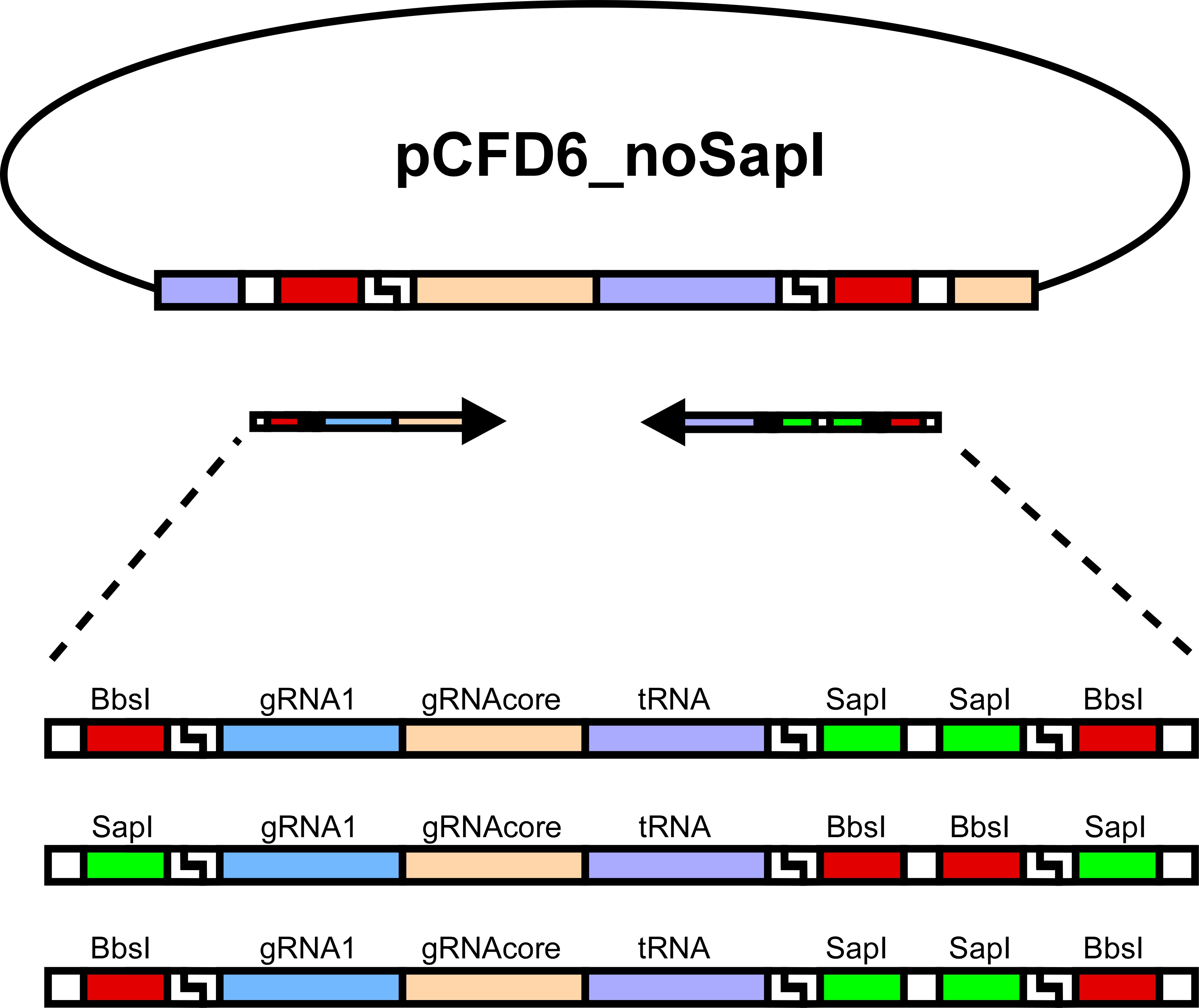

Supplement: Supplementary file 1 [file metabolites-12-00315-s001.zip › metabolites-1660805-supplementary/Supplementary Figure S9.jpg]
